# Manipulation of single cells via a Stereo Acoustic Streaming Tunnel (SteAST)

**DOI:** 10.1038/s41378-022-00424-9

**Published:** 2022-08-04

**Authors:** Yang Yang, Wei Pang, Hongxiang Zhang, Weiwei Cui, Ke Jin, Chongling Sun, Yanyan Wang, Lin Zhang, Xiubao Ren, Xuexin Duan

**Affiliations:** 1 State Key Laboratory of Precision Measuring Technology and Instruments, Tianjin University, Tianjin, 300072 China; 2 Tianjin Medical University Cancer Institute & Hospital, Tianjin Medical University, Tianjin, 300072 China

**Keywords:** Engineering, Physics

## Abstract

At the single-cell level, cellular parameters, gene expression and cellular function are assayed on an individual but not population-average basis. Essential to observing and analyzing the heterogeneity and behavior of these cells/clusters is the ability to prepare and manipulate individuals. Here, we demonstrate a versatile microsystem, a stereo acoustic streaming tunnel, which is triggered by ultrahigh-frequency bulk acoustic waves and highly confined by a microchannel. We thoroughly analyze the generation and features of stereo acoustic streaming to develop a virtual tunnel for observation, pretreatment and analysis of cells for different single-cell applications. 3D reconstruction, dissociation of clusters, selective trapping/release, in situ analysis and pairing of single cells with barcode gel beads were demonstrated. To further verify the reliability and robustness of this technology in complex biosamples, the separation of circulating tumor cells from undiluted blood based on properties of both physics and immunity was achieved. With the rich selection of handling modes, the platform has the potential to be a full-process microsystem, from pretreatment to analysis, and used in numerous fields, such as in vitro diagnosis, high-throughput single-cell sequencing and drug development.

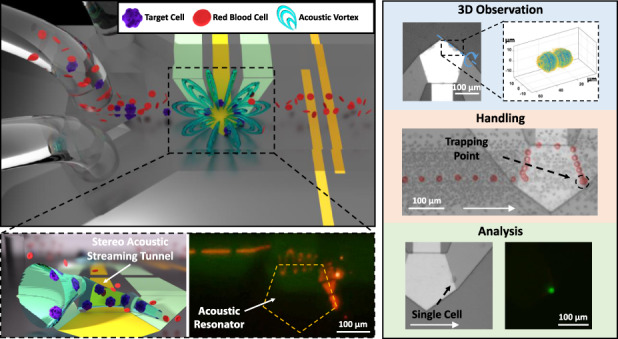

## Introduction

Research in single cells is a rapidly growing field where heterogeneous cellular characteristics, such as morphology^1^, adhesion^2^, mobility^3^, protein expression^4^ and gene expression^5^, are assessed on the basis of individual cells. The fundamental advantage of single-cell analysis methods over bulk assays is that retaining single-cell information can reveal rare cell properties and biologically meaningful heterogeneity between individual cells. The preparation and manipulation of individual cells, including dissociation^6^, trapping^7,8^, rotation^9,10^, staining^11^, release^12^, and pairing^13^, are essential capacities in biotechnology that are fundamental for various purposes, such as single-cell analysis^3,4,9,14^, drug development^15^, organ-on-chip systems^16^ and cell–cell interaction studies^13^.

Microfluidics-based methods are a highly effective strategy to achieve single-cell-level manipulations, where the dimensions of force gradients and physical features are on the same scale as individual cells. When dealing with complex biological samples, such as blood^17,18^, sputum^19^ and stool^20^, a micro total analysis system (μ-TAS), which can be integrated with multifunctional modules, has shown excellent compatibility and performance. Affinity capture is a commonly used strategy based on affinity ligands, such as antibodies and aptamers, modified on microstructures^12^. Although this method has demonstrated excellent performance in capture efficiency and specificity, it is inferior to the physical characteristics-based methods in terms of versatility and flexibility. Several techniques, divided into passive and active strategies, have been established. Hydrodynamic methods passively guide individual cells in continuous flow to design microstructures that achieve single-cell trapping^13,21–25^. Regarding active methods, a number of techniques have been used, including dielectrophoresis^26^, optical tweezers^27,28^, magnetic tweezers^14,29–31^, and acoustophoresis^32–39^. Among these technologies, acoustic-based strategies have received considerable attention due to their biocompatibility, flexibility and low cost^40–44^. Recently, a new method based on acoustic streaming, which realizes the handling of particles by weakening the effect of acoustic waves and amplifying the effect of acoustic streaming, has been established for motion^45,46^, enrichment^47^, selective trapping^48–52^, and rotation of microscale specimens^9,46^. Compared with conventional acoustophoresis-based strategies, acoustic streaming provides more dynamic conditions, which significantly improves the ability to manipulate and analyze samples^41,47,53^. Although many breakthroughs in the observation, spatial movement and interaction of single cells have been achieved, there are still barriers between the current handling modes and complex requirements in single-cell research, for example, three-dimensional (3D) observation without fluorescent labels and dissociation of doublets. In addition, integrating these handling modes (rotation, dissociation, separation, and analysis) on one chip is still a challenge, especially when specific operations often require multiple valves and pumps to control the transportation of fluids or substances.

**Fig. 1:**
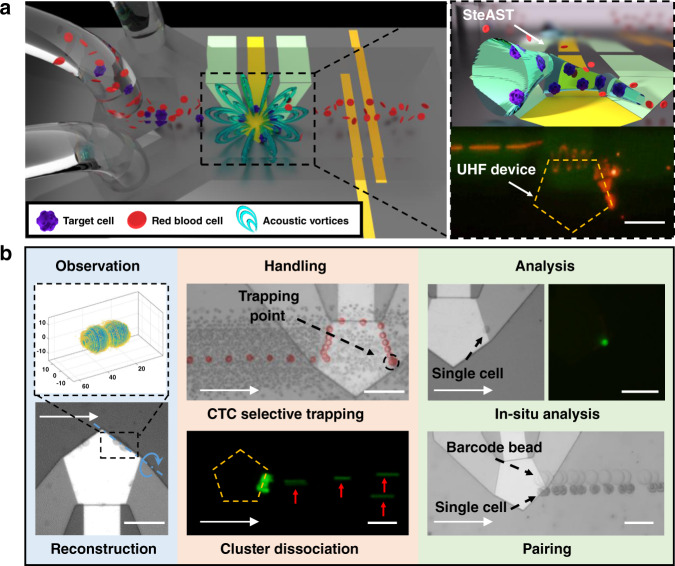
Schematics and multimode manipulation of the SteAST platform. **a** A schematic of the SteAST platform. The SteAST platform comprises a microfluidic channel bonded to a UHF BAW resonator on a silicon substrate. The attenuation of BAWs in a coupled liquid triggers 3D acoustic streaming vortices. The detailed images show a cartoon profile of SteAST and the stacked image that demonstrates the trajectory of a fluorescent particle (5 μm) in the tunnel to describe the actual profile of SteAST. **b** SteAST-based multimode manipulation of single cells. The reconstruction of the cluster was achieved via rotation manipulation, demonstrating successful 3D observation. As a demonstration of the pretreatment of biological samples, this platform provides size-based selective trapping of a single cell and shear force-based dissociation of clusters. To show successful analysis, in situ analysis of single cells by staining and pairing a target cell with a barcode gel bead for downstream analysis was demonstrated with the cooperation of the customized microfluidic channel. White arrows show the direction of lateral flow. The blue dotted line and blue arc arrows show the axis and direction of rotation, respectively. The red arrows point to the individual cells after dissociation. The scale bar is 100 µm.

In this study, we utilized an ultrahigh-frequency bulk acoustic wave device (UHF BAW device) to create 3D acoustic streaming, called stereo acoustic streaming (SteAS), which is highly confined by a microchannel to form a virtual tunnel distributed along the boundary of the device. “Stereo” is the core feature of our technology that differentiates it from the classic acoustic streaming-based acoustofluidic technologies^49,54,55^, which means the acoustic streaming in our device is three-dimensionally distributed in the microfluidics and the particles/cells are trapped into a fixed trapping point (0-dimension) with full spatial confinement. Cells in the stereo acoustic streaming tunnel (SteAST) were arranged and migrated along the tunnel with a spiral trajectory, as shown in Fig. 1a. In previous studies, although SteAS has been developed for particle enrichment, the potential for handling biological specimens and the complexity of the spatial distribution of acoustic streaming vortices have not been exploited. By understanding the relevant forces and optimizing the boundary conditions, a virtual tunnel whose diameter matched the size of single cells was generated. The migration of cells in the tunnel is the result of the combined and competing effects of lateral flow, acoustic streaming, and acoustic waves and interactions. Multiple effects were utilized to realize the multimode manipulation of individual cells in one chip, including rotation, dissociation, selective trapping, controllable release and particle pairing. Based on these modes, a variety of paradigms from observation to analysis were achieved, as shown in Fig. 1b. Moreover, to demonstrate the system’s application in clinical and biological research, we proposed a strategy to separate and in situ analyze circulating tumor cells (CTCs), a kind of rare cell in cancer patient blood, from patients’ undiluted blood through the integration of these operations. To the best of our knowledge, there is no existing microfluidic device that has established the capacity to achieve such complex and precise manipulation of single cells. We believe that the SteAST platform, which provides a brand-new method for the multimode manipulation of single cells, will benefit various biomedical and biological applications.

## Results

### Working principle and design of the SteAST system

**Fig. 2:**
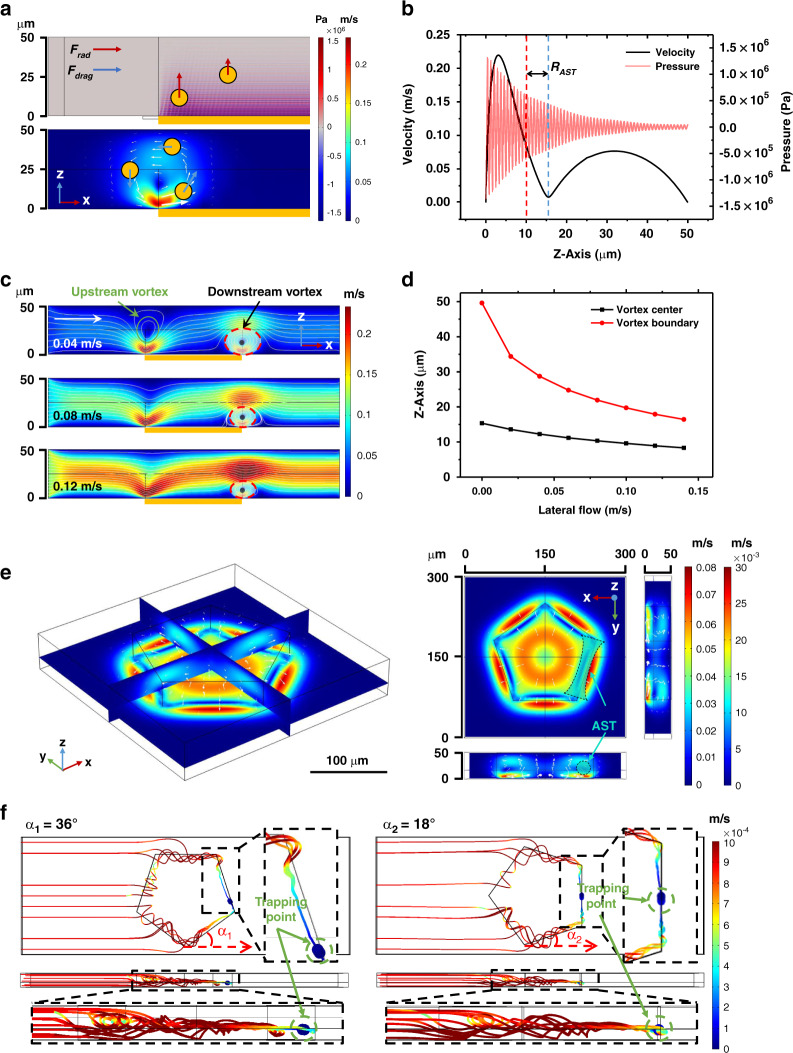
Design of the SteAST platform. **a** Simulation results of the distribution of the acoustic field (1.8 GHz) and acoustic streaming in the microchannel. The acoustic radiation force and drag force on particles are highlighted by red and blue arrows, respectively, in the SteAS platform. The length of the arrow represents the magnitude of force. The position of the UHF BAW device is represented by yellow rectangles. **b** Distribution of the acoustic field and velocity of acoustic streaming along the height in a 50 μm high microchannel. *R*_*AST*_ is the inside conduit radius of the AST. **c** The distribution of the flow field in the microchannel under lateral flow at different velocities (0.04, 0.08, and 0.12 m/s). The white arrow points in the direction of lateral flow. The position of the device is shown by yellow rectangles. The upstream and downstream vortices are highlighted by green and black arrows, respectively. The center and boundary of the downstream vortex are represented by black points and red dotted cycles, respectively. **d** The height of the center and boundary of the acoustic streaming vortex at the downstream boundary of the device with lateral flow at different velocities. **e** The 3D simulation result of the SteAST field generated by the pentagonal UHF device under the restriction of the microchannel. The color bar on the left corresponds to the velocity in the x-y plane, and the color bar on the right corresponds to the x-z and y-z planes. The height of the image in the x-y plane is 17 μm, which is approximately one-third the height of the microchannel (50 μm). The AST is highlighted by cylinders and cycles in the top and side views, respectively, as indicated by the cyan arrows. The center and wall of the AST are the low-velocity and high-velocity zones in SteAS, respectively. **f** The trajectories and velocity spectrum of particles trapped by SteAS under different angles between the UHF device and lateral flow, which is defined by α. When α is 36°, the trapping point is biased toward the vertex near the downstream, and when it is 18°, the trapping point is at the center of the tunnel.

To fulfill the requirements of single-cell manipulations, the SteAST system was designed by integrating a UHF BAW device into a microfluidic channel. The geometry of the channel and the shape, position and alignment of the device in the microchannel were optimized to create a tunable 3D AST, which is discussed thoroughly in this section (Fig. 2) and functions as a dynamic single-cell-sized potential well (Fig. 1a). This is the core design to achieve size-selective and tunable cell trapping. Cell trapping was achieved due to the balance between the strength of SteAS and lateral flow, which can be controlled by tuning the power applied to the BAW device and the lateral flow rate. This results in a dynamic cell manipulation system, where cell trapping, solution exchange, on-site cell analysis, and cell release can be well controlled under continuous flow conditions without the use of extra valves. In addition, this platform could provide a stable and adjustable shear force to dynamically trap cells by optimizing the spatial position of the tunnel and lateral flow parameters. As a proof of concept, manipulations including cell rotation, cluster dissociation, cell reconstruction, and cell-particle assembly were demonstrated with the SteAST system (Fig. 1b).

First, we discuss the details of the working and design principles of the SteAST system. The optical image, SEM image and cartoon section view of the GHz BAW device are shown in Supplementary Fig. S1a–c. The device is a typical thin film piezoelectric resonator that contains a Bragg mirror structure, a bottom electrode, a piezoelectric layer and a top electrode. The resonant frequency of the device is shown in Supplementary Fig. S1d. The mechanical displacements result in a standing acoustic field in the body of the piezoelectric layer by applying resonant frequency signals across the piezoelectric layer (Supplementary Fig. S1e). As the BAW device is placed in direct contact with the liquid, efficient acoustic energy coupling from the piezoelectric layer to the surrounding liquid is guaranteed. The simulation results of the distribution of the acoustic waves at 2 GHz and 200 MHz are shown in Supplementary Fig. S2a. The difference in the distribution and decay length of the acoustic waves at these two frequencies can be clearly observed. The nonlinear attenuation of oscillating displacements in dispersive media results in a body force (*F*_*B*_) at the z-axis, which pushes the liquid in the direction of acoustic wave propagation and then generates a stable liquid flow (acoustic streaming)^56^. Since *F*_*B*_ ∝ *ω*^*4*^ (*ω* is the angular frequency), the strength of *F*_*B*_ generated by UHF BAWs is much stronger, which is due to the enhanced local energy density^57–59^. In addition, the rather small footprint of the UHF device (dozens to millions of square microns) results in a more focused acoustic wave beam^60^. As *β*^*−1*^ ∝ 1/*ω*^*2*^ (*β* is the attenuation coefficient and *β*^*−1*^ is the attenuation length in liquid media), the decay length of the acoustic wave is actually less than 20 μm. Thus, there are almost no standing acoustic waves in the SteAST system.

Due to the particularity of the thickness extension (TE) vibration mode and high resonance frequency, the fluid jets rise from the top of the device and impinge on the top interface of the microchannel; they recirculate vigorously in a clockwise or counterclockwise direction to form hourglass-shaped stereo acoustic streaming vortices, which behave like microscale fountains. As the microvortices are connected, a series of closed microvortices form a virtual tunnel, whose contour profile is defined by the shape of the resonator (Figs. 1a, 2a and S2b). Such a virtual tunnel is the basic component of the SteAST system. We used finite element simulation software (COMSOL) to discuss the influence of different boundary conditions to optimize the SteAST platform. First, we considered the particles in the system without lateral flow. As shown in Fig. 2a, they experienced an acoustic radiation force (*F*_*rad*_) and a Stokes drag force (*F*_*drag*_), which are induced by 3D acoustic streaming vortices. A 2D simplified model was used here based on the symmetry of the acoustic streaming pattern. The acoustic field was distributed above the device, and the microvortices levitated around the boundary of the device. The acoustic radiation force pushed the particles above the device away along the z-axis, while the drag force drove the particles to move along the vortex streamline. Ideally, under the combined forces, the particles will be translated along a spatial trajectory and trapped in the low-velocity area, which is the equilibrium position at the center of the vortex.

Tuning the inside conduit diameter of the tunnel to fit the size of a single cell is the 1st design principle of the SteAST platform. This can be achieved by adjusting the boundary conditions of the acoustic streaming. An acoustic radiation force is triggered by the scattering of acoustic waves from the BAW device. In the case where the particle is smaller than the characteristic length scale of a nonuniform acoustic field, the time-averaged radiation force exerted on a spherical specimen can be expressed as^61,62^

$$\begin{array}{l}{\it{F}}_{{\it{rad}}} = - \nabla {\it{U}}\\ {\it{U}} = \frac{{4{\it{\uppi }}}}{3}{\it{r}}^3\left[ {{\it{f}}_1\frac{{{\it{\upkappa }}_{{{\boldsymbol{l}}}}}}{2}\left\langle {{\it{p}}_{{\it{in}}}^2} \right\rangle - {\it{f}}_2\frac{{3{\it{\uprho }}_{\it{l}}}}{4}\left\langle {{\it{v}}_{{\it{in}}}^2} \right\rangle } \right]\\ {\it{f}}_1 = 1 - \frac{{{\it{\upkappa }}_{\it{p}}}}{{{\it{\upkappa }}_{\it{l}}}}\\ {\it{f}}_2 = \frac{{2\left( {\frac{{{\it{\uprho }}_{\it{p}}}}{{{\it{\uprho }}_{\it{l}}}} - 1} \right)}}{{2\frac{{{\it{\uprho }}_{\it{p}}}}{{{\it{\uprho }}_{\it{l}}}} + 1}}\end{array}$$

where *U* is the force potential field, which is determined by the particle parameters and characteristics of the fluid; *r* is the particle radius; *f*_*1*_ and *f*_*2*_ represent the monopole and dipole scattering coefficients, respectively, which relate to the bulk and directional vibration in an oscillating field; *κ*_*p*_ and *κ*_*l*_ are the compressibilities of the particle and liquid, respectively; *ρ*_*p*_ and *ρ*_*l*_ are the densities of the particle and liquid, respectively. The equation indicates that the radiation force on the particle is determined by the spatial gradient of the force potential field, which is proportional to *r*^3^. Thus, the acoustic radiation force is strongly dependent on the distance from the surface of the BAW device and the radius of the particle. The Stokes drag force induced by acoustic streaming can be estimated as

$${\it{F}}_{{{{\boldsymbol{drag}}}}} = 6{\it{\uppi \upmu rv}}$$

where *μ* is the dynamic viscosity of the medium and *v* is the nonoscillatory velocity of the particle relative to the liquid.

Although the relevant factors of the force are clear, quantitative calculations of the radiation force applied to the trapped particles are rather difficult since it is highly related to the spatial position of the particle. Here, we qualitatively discuss the influence of the boundary conditions and the force status of the trapped particles by simulation. Once the device was fixed, the strength and distribution of the acoustic streaming were mainly determined by the geometry of the microchannel^59,63,64^. We then calculated the distribution of the acoustic field in 2D models under different geometric confinements, as shown in Supplementary Fig. S3. Since the width of the microchannel is often larger than its height, the model was simplified as being confined at the top. Supplementary Fig. S4 shows the acoustic pressure at the center and velocity of the acoustic streaming on the boundary of the UHF device confined by different heights. When the height of the flow channel is larger than 25 μm, the distribution of acoustic waves is dominated by the attenuation effect and is no longer related to the height of the flow channel (reflection effect). Correspondingly, when the height is less than 25 μm, the velocity of the acoustic streaming decreases, which indicates that the acoustic energy cannot be completely converted into fluid kinetic energy. Therefore, to obtain the ideal intensity of acoustic streaming while avoiding the negative effects of the standing waves, the height of the microchannel should be higher than 25 μm. In addition, when the height is higher than 100 μm, the acoustic waves cannot reach the center of the vortex, which is the point of minimum velocity (at approximately one-third in the z-axis; shown in Supplementary Figs. S3 and S4). Therefore, the radiation force is not strong enough to push the particles into the vortex center, which manifests as the expansion of the inside conduit diameter of AST. Therefore, a microchannel with a height of 50 μm was chosen as an ideal boundary condition for generating the right AST targeting for single-cell manipulations. In this case, the acoustic waves can cover the center of the acoustic streaming vortex and create a single-cell-sized potential well at one-third of the height of the microchannel (Fig. 2b). The center of the SteAST is the center of the acoustic streaming vortex marked by the blue line, and the boundary of the SteAST is determined by the position where the *F*_*rad*_ cannot further drive particles away from the device. Since *F*_*rad*_ is related to the particle size, acoustic properties and sound pressure, it is difficult to give an accurate boundary; here, we define the boundary as the position where the sound pressure decays rapidly, which is marked by a red line. In the actual experiment, the applied power can be adjusted to precisely tune the boundary. Again, tuning the geometric confinement of the microchannel to achieve the right conduit diameter of AST is the 1^st^ design principle of the SteAST system for accurate and stable manipulation of single cells.

Then, we consider the lateral flow effect. From an energy point of view, the particles trapped in the AST are affected by the kinetic energy of the lateral flow. When the kinetic energy exerted on the particles is higher than the energy barrier of the potential well induced by acoustic waves and vortices, the particles will leave the tunnel and move downstream along with the lateral flow. Notably, the trapping stability can be dynamically tuned on demand by changing the power applied to the device and the flow rate, thus enabling a versatile cell manipulation tool, where trapping, reaction, incubation and analysis of the cells can be achieved on the same chip under continuous flow conditions. The balance between trapping and releasing represents the 2nd design principle.

Next, we discuss the details of the fluid interactions between the lateral flow and acoustic streaming based on a 2D model. Due to the particularity of the TE mode of the BAW device, the jetting flow and lateral flow form an orthogonal relationship in a highly confined environment (Fig. 1a); thus, the symmetric fountain-like acoustic streaming vortices could be affected by the lateral flow according to their spatial position with the flow direction. As shown in Fig. 2c and Supplementary Fig. S5, due to the interactions with the lateral flow, the upstream vortex will be lifted while the downstream vortex will be pressed down; thus, asymmetric acoustic streaming vortices result in the x-z plane. To further analyze the effects of asymmetric vortices on particle trapping, we calculated the velocities of the upstream and downstream vortices. As shown in Supplementary Fig. S6a, by fixing the power applied to the device, the upstream vortex moves away from the device center and continues to weaken until it disappears as the lateral flow rate is increased. This indicates that cells are more difficult to capture at a high lateral flow rate since the trapping point (center of the acoustic streaming vortex) moves away from the acoustic radiation force area. For the downstream vortex, the velocity of the reflux fluid below the equilibrium position is slightly suppressed as the lateral flow rate increases, while the flow velocity above the flow channel increases rapidly (Supplementary Fig. S6b). The flow above the equilibrium position has both a vortex part that transports particles back to the acoustic radiation force region and a lateral flow part that takes the particles downstream. To decouple the two parts, we extracted the height of the vortex center and the upper boundary of the vortex at different flow rates, which is displayed in Fig. 2d. The boundary of the vortex is calculated based on the conservation of flux in the vortex section. This shows that as the lateral flow velocity increases, the height of the vortex center and the boundary both decrease, and the latter decreases faster. This brings about two effects: one is that the capture position approaches the device, which will result in an increase in the acoustic radiation force by pushing the particles out of the vortex center. The other effect is the shrinking of the AST area and expansion of the lateral flow area. The AST and lateral flow are actually in a competitive relationship. Thus, the weakening of the former corresponds to the enhancement of the latter, which will reduce the trapping stability. Especially when the particle size is larger than the size of the shrinking AST, the particles will inevitably be directly affected by the lateral flow, resulting in the failure of trapping.

From this part of the analysis, the trapping status of the particles (trapping or releasing) can be controlled by tuning the energy balance between the lateral flow and acoustic streaming. Specifically, their interactions result in an asymmetric vortex pattern, which will further affect the performance (capture efficiency, trapping stability, etc.). Taking into account the transport effect of lateral flow and the stability of the vortex, the flow rate needs to be optimized to ensure that all the particles will be deflected by the upstream vortex, which is the prerequisite for trapping in the downstream vortices.

After understanding the fluidic channel confinement and lateral flow effects on particle trapping, we further considered the arrangement of the AST and its effects on particle behaviors, which gives the 3^rd^ design principle of the SteAST. The shape of the device determines the profile of the AST. In this work, we take the best performance (quality factor, resonant frequency) as the priority principle of the device design; thus, a pentagonal UHF device with an area of 20 k μm^2^ was used in this system^65,66^. Here, a 3D simulation model was applied to deeply analyze the interactions between lateral flow and the AST in 3D space. As shown in Fig. 2e, a pentagonal-shaped AST was clearly observed, which exactly followed the periphery of the device. Once the shape of the device is fixed, the relative arrangement of the device with the microchannel will affect the behavior of particles in the AST, which in turn affects its trapping performance.

As discussed above, in a stable trapping process of SteAST, all the particles were deflected by the upstream vortices first and then passed through the AST before reaching the end trapping position. To further analyze the migration process of particles in the AST, the velocity spectrum and trajectories were thoroughly analyzed (Fig. 2f). The particles were initially evenly distributed in the microfluidic channel before meeting the front vortex. From the top view, after contact with the front vortices and being dragged by the lateral flow, the trajectories of particles were only distributed on the boundary of the UHF device, where the AST sits. In the end, they were statically trapped at one point in the tunnel (the trapping point), which is highlighted in Fig. 2f. From the side view, it is noted that the particles were also focused in the z-axis at the center of acoustic streaming vortices without contact with the substrate. This analysis gives a clear picture of the trapping process. Once in contact with the front vortices, the particles entered the AST and moved downstream under the action of the drag force induced by lateral flow while being concentrated at the equilibrium position. When the particles arrived at the boundary downstream, regardless of the initial position of the particles, all the particles were concentrated in the center of the tunnel, which provides a 3D focusing condition, as shown in the detailed image in Fig. 2f and SI-Movie-1. It is worth emphasizing that, unlike trapping through an individual potential well, SteAST can decrease the velocity of the particles through continuous hydrodynamic action before entering the stable trapping point, which we call the buffering effect. 3D focusing combined with the buffering effect provides a unique and reliable dynamic trapping strategy. Next, we discuss the influence of the angle between the device and the lateral flow on the particle trajectory. As shown in Fig. 2f and SI-Movie-2, the final trapping position in the downstream boundary actually moves with the angle between the flow direction and device, which is defined by the angle α. The simulation results show the trapping process with α values of 36 degrees and 18 degrees and the migration of trapping points. Due to the poor continuity of the AST at the apex of the device, the trapping point near the apex is unstable. Based on this, when dealing with complex samples with strong interactions between particles, we can tune the relative angle to move the trapping point away from the vertex to achieve better performance. This part will be further discussed in the experimental part of the selective cell trapping in undiluted blood.

### Quasi-static trapping and 3D reconstruction of a cell cluster

After establishing the design principles, we tested the SteAST system for cell trapping. First, cell trapping without lateral flow was tested. Unlike the capture based on microstructures, the cells trapped by SteAS are not stationary in the AST, but they rotate along the direction of the vortex streamline on the axis of the equilibrium position. This phenomenon is called quasi-static trapping.

**Fig. 3:**
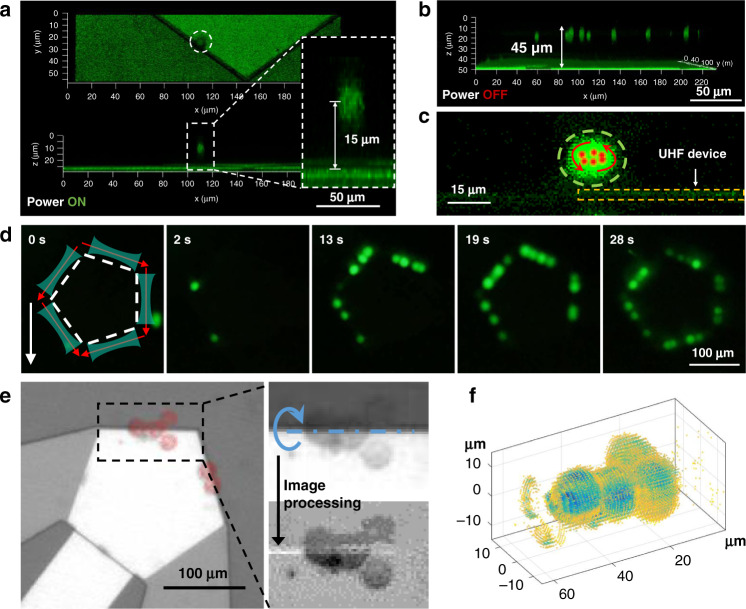
Quasi-static trapping and 3D reconstruction of cells. **a** SteAS-based trapping of a single fluorescent PS particle (5 μm). When the power was applied, particles are trapped and suspended in the microchannel. **b** When the power is turned off, the particles settle to the surface. **c** A suspended particle is trapped and rotated in the AST under the combined effect of radiation force and drag force. The stacked image sequences (6 images, 27 ms apart) are captured at approximately 10 mW. The green dotted circle represents the range of trapping points. The red arrows show the direction of particle rotation. The yellow dotted rectangle shows the position of the UHF device. **d** Pattern of individual cells based on the AST. HeLa cells stained with calcein-AM (green) are used to represent the size and profile of the trapping point. The white arrow represents the direction of lateral flow, the red arrows show the trajectories of individual cells in SteAS, the white dotted pentagon is the position of the UHF device, and the blue curved rectangles represent the profile of the AST. **e** A single cluster trapped in the AST. The cells are highlighted by pseudocolor (red). The detailed images show the images before and after image processing. The rotation axis and direction are represented by a blue dotted line and a blue arrow, respectively. **f** The result of 3D reconstruction.

According to the 1st design principle, we used a microchannel with a height of 50 μm. To better demonstrate the actual working state of the SteAST system, confocal microscopy (Leica, Germany) and 5 μm fluorescence polystyrene (PS) beads were used to characterize the spatial location and size of the AST. When power was applied, particles were observed to be stably trapped and suspended in the microchannel, which was approximately 15 μm above the device, as shown in Fig. 3a and SI-Movie-3. The trapping positions exactly match the equilibrium positions in the simulations (Fig. 2). After the power was turned off, the particles moved up to 45 μm due to the combined action of buoyancy and gravity, as shown in Fig. 3b. To further observe the trajectory of a particle and the extent of the equilibrium position, the x-z-t mode of the confocal microscope was applied, where the imaging speed reached 37 frames per second. A composite stacked image (6 images, 27 ms apart) is shown in Fig. 3c. The original images and the videos are shown in Supplementary Fig. S7 and SI-Movie-4. The red point indicates the center of the particle in each frame, the green dotted circle represents the range of particle motion, and the red arrows indicate the direction of the particle motion. The result demonstrates that the particle was faithfully trapped in the tunnel. It is noted that the diameter of the equilibrium position is approximately 15 μm, which is at the same scale as a single cell. This provides the key prerequisite for precise single-cell manipulation. This also proves that SteAST-based cell manipulation is a contactless process, which is essential for the maintenance of cell viability and prevention of contamination issues.

Next, the trapping of individual HeLa cells was demonstrated. As mentioned before, the vortices induced by the UHF BAW were connected to adjacent vortices and assembled into an AST along the boundary of the device. The results shown in Fig. 3d and SI-Movie-5 illustrate that the individual cells were trapped in the equilibrium position and patterned as the shape of the device, which means that SteAS creates a suspended trapping tunnel where its conduit diameter is comparable to the single cell size and profile is determined by the shape of the device.

Interestingly, the trapped cells are rotated in the AST. This is due to the shear effect of the acoustic streaming vortices acting on the cell surface; a torque is generated, which makes the spatial position of trapped cells stationary, but rotation occurs. Therefore, the axis of the cell rotation is the axis of the tunnel, and the direction of rotation is the direction of the vortices (Fig. 3e). Based on this behavior, we then realized the 3D observation of cells or clusters.

Spatial localization is a key determinant of cellular behavior and an important parameter to understand heterogeneity and cell–cell interactions in clusters, tissues and organs. The most common method for obtaining spatial information is scanning confocal microscopy, which realizes 3D reconstruction by rebuilding fluorescent image slices. Using out-of-plane rotational manipulation to observe 3D spatial information is an alternative method. Although single-cell-level rotational manipulation has been reported for rotating C. elegans^9,67^, rotational manipulation and reconstruction for irregularly shaped cell clusters without fluorescent labels has remained a significant challenge. To achieve reconstruction, movement in the x-y plane should be suppressed, the rotation axis must be fixed without waggling, and the speed of rotation should be tunable and sufficiently uniform. These requirements are well met by the SteAST platform. A high-speed camera (Photron, Japan) was used to capture the process and state of cells trapped in the AST. A single cell and a dimer of HeLa cells were utilized to evaluate the stability of the SteAS-based rotational manipulation, as shown in Supplementary Fig. S8 and SI-Movie-6. The image sequences showed that the cells were strictly confined in the AST without movement and rotated at a uniform speed in a complete rotation cycle. The speed of rotation can be adjusted by tuning the applied power, and the relationship between them is shown in Supplementary Fig. S9. Moreover, a cell tetramer was also chosen as a demo of a cluster for 3D reconstruction. The trapped cluster was suspended and rotated in the out-of-plane direction, as shown in Fig. 3f. Since the profile of the SteAST is patterned by the edge of the UHF device, there are gray zones (silicon) and white zones (gold) in the background of the image. To obtain a better background image, we processed the original image, and the images before and after processing are shown in detail in Fig. 3e. The processes are demonstrated in Supplementary Fig. S10. Similar to the strategy of reconstruction based on layer-layer slice images in a Cartesian coordinate system through positioning by height information, 3D reconstruction can be performed in a polar coordinate system based on rotational slice images through positioning by angle information. The interval angle among image sequences was calculated by dividing a period (2π) by the number of pictures in a period to determine the relative position of each picture in a polar coordinate system. The reconstructed graphics can be obtained through coordinate transformation, as shown in Fig. 3f. The image processing and 3D reconstruction were achieved by MATLAB software (USA).

### Dynamic cell trapping and quantum release

After achieving cell trapping with the SteAST system under static conditions, we then demonstrated cell trapping under lateral flow conditions. According to the 2^nd^ design principle, the balance between the AST and lateral flow will determine the cell trapping status, and the transportation and shearing effects of lateral flow actually provide more possibilities for cell manipulations in this platform. The SteAS-based trapping of cells in continuous flow is called dynamic trapping. Here, “dynamic” has two meanings. The first is that selective trapping or controllable release can be achieved through the dynamic adjustment of lateral flow and acoustic streaming; the second is that the specimens or reagents transported by lateral flow can be dynamically controlled in time and space to meet the complex requirements in different applications. In this part, we first verified the buffering effect during cell trapping by analyzing the trajectories of the cells. Selective cell trapping and controlled release are demonstrated, and the relationship between the number of captured cells and the lateral flow effect is emphasized.

**Fig. 4:**
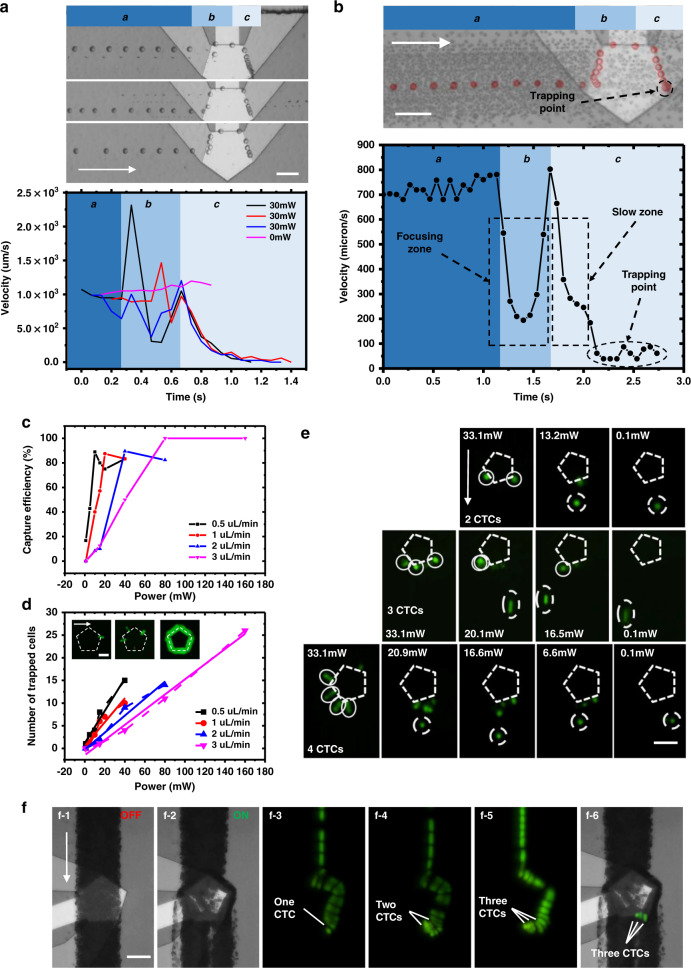
Dynamic cell trapping and size-based separation. **a** The stacked images demonstrate the trapping process of the single cell in different initial positions via SteAST. The graph shows the velocity spectrum of individual cells in the trapping process. The trapping process is divided into three stages: the lateral flow zone (region (a)), focus zone (region (b)), and buffering zone (region (c)). The cells are finally captured at the trapping point. **b** The trajectory of trapped single HeLa cells in diluted blood. The stacked image is created by merging 41 frames. The graph shows the velocity spectrum of trapped single HeLa cells. **c** Capture efficiency of HeLa cells stained with calcein-AM (Invitrogen, USA) at different applied powers and flow rates. The fluorescence field was observed, the number of cells trapped by SteAST and the number of cells flowing through were counted, and the capture efficiency was calculated based on the ratio of the two. **d** The relationship among the number of trapped cells (capture capacity), applied power and flow rate. The images show the expansion of the trapping position as the power increased. **e** Digital release of the trapped cell. Multiple cells (2, 3, 4 HeLa cells dyed by calcein-AM) are trapped in SteAST, and the digital controllable release of the individual cells one by one is achieved by gradually reducing the power to regulate the capturing capacity. **f** Selective trapping of HeLa cells (CTCs) in undiluted whole blood. (f-1) Hydrodynamic focusing of a whole blood sample. (f-2) Blood cells release when the power is turned on. (f-3) ~ (f-5) Selective trapping of individual CTCs. The stacked image sequences demonstrate the trajectories of trapped CTCs. (f-6) The merged image shows that the three CTCs were stably trapped while the blood cells were released from SteAST. The ratio of double focusing fluid and cell fluid is 1:1:1. The total flow rate was 1 µL/min. The scale bars are 100 μm.

To analyze the process of cell trapping under lateral flow conditions, individual HeLa cells were injected into the microchannel as tracking particles, as shown in Fig. 4a and SI-Movie-7. The stacked images demonstrated the process of dynamic trapping of three individual cells in different initial positions. The velocity spectrum of the cells was calculated by the position and interval time of the cell in the adjacent images. We divided the trapping process into three phases that occur in regions (a)~(c). In region (a), the cells move at a uniform speed in the lateral flow. In region (b), the cell meets the front microvortex, where their flow direction and velocity are changed dramatically. As the trapped cells are dragged into the AST, they move along a fixed pathway (the boundary of the UHF device) while being 3D focused in the channel. The velocity of the cell is chaotic in region (b) due to the randomness of the initial position of the cells; however, after going through 3D focusing, they all enter the next area at the same speed and trajectory. Here, we refer to region (b) as the focusing zone. In region (c), the velocity of the cell decreases gradually until it arrives at the trapping point. Thus, region (c) is called the buffering zone. At the trapping point, the cell is trapped in a fixed position where the drag force and acoustic radiation force balance each other. The experimental results are rather consistent with the simulation, which proves the complexity of dynamic trapping based on SteAST. Compared with other 2D-based trapping, 3D focusing and buffering effects make this platform more robust and widely applicable.

Next, we discuss the size-selective cell trapping enabled by the balance between the drag and radiation forces. Based on the theoretical discussion, the trapped cells in the AST were affected by both drag and radiation forces. Due to *F*_*rad*_*/F*_*drag*_ ∝ *r*^*2*^, larger specimens are displaced more substantially than smaller specimens; thus, larger specimens are trapped in the center of vortices, while smaller specimens leave the AST under the action of lateral flow, which is the theoretical basis of size-based selective trapping. This theory was verified through both simulations and experiments, as shown in Supplementary Fig. S11 and SI-Movie-8. A 3D simulation model was built to analyze the trajectories of particles with different sizes. The red particles (15 μm) were trapped at the boundary of the device, while blue particles (2 μm) escaped the trap. The experimental results of selective trapping of PS particles (5 μm and 2 μm) are shown in Supplementary Fig. S11c. We speculated that the resolution of separation in the simulation was worse than that in the actual experiment since their interactions were totally ignored in the simulation model. In real situations, smaller particles have fewer chances to enter the AST as larger particles enter the trapping position much faster. In addition, since larger particles could be more stably trapped, smaller particles in the potential well would be easily squeezed out and replaced by larger particles.

To further observe the dynamic selective trapping process in the presence of cell interactions, HeLa cells were spiked into diluted whole blood to represent a complex biological sample. The trajectory of the trapped HeLa cells was demonstrated in the composite stacked image, and the velocity spectrum is shown in Fig. 4b. As explained in the previous discussion, the cells underwent the 3D focusing and deceleration process in the focusing and buffering zones. Due to the smaller size of the red blood cells, the radiation force was not strong enough to confine them within the AST, and the hydrodynamic force dominated, which resulted in their passing through the device downstream without being trapped. Thus, the SteAST system provides a versatile size-based cell separation system by trapping larger cells while ignoring smaller cells. The capture efficiency is the key parameter for such a cell separation platform. The capture efficiency results with different applied powers and lateral flow rates are shown in Fig. 4c. The capture efficiency increased with applied power and decreased with increasing lateral flow rate. The reason is that the increasing lateral flow triggers a stronger shear force and decreases the stability of the AST, while the increasing applied power generates acoustic streaming vortices at a higher velocity and impairs the lateral flow, which is explained in Fig. 2. To create a stable trapping point with high resolution, the applied power and lateral flow rate should be well balanced. Our platform can maintain the capture efficiency at a high level with a lateral flow rate from 0.5~3 µL/min. It should be noted that although high power leads to high capture efficiency, it sacrifices the purity of trapped cells because smaller cells will be mistakenly trapped as well. In addition, to ensure that the cells in the lateral flow pass through the SteAS area, we calculated the actuating range of acoustic streaming under different power and flow rates, called the capture range, as shown in Supplementary Fig. S12. One side of the UHF device was parallel to the direction of lateral flow, called the target side. The boundary of the fluorescent fluid (rhodamine B) overlapped with the target side through the sheath flow, and the capture range was calculated by measuring the length between the target side and the boundary of the dye, which was transported by acoustic streaming vortices. Then, the introduction of focus by sheath flow strictly limited the sample flow within the capture range to improve the efficiency.

Beyond trapping, release is a necessary step for downstream in-depth cell analysis. However, controlled release is still a challenge due to the inherent problems of separation strategies, such as surface adhesion or microstructure-based capture^12^. Moreover, the retrieval process is often random, as the local flow in the microfluidic chip is difficult to precisely control and cell-to-interface adhesion may occur randomly^68–71^. Controlled cell release has been largely studied using active approaches by applying changing magnetic^3^, dielectric^72^ or acoustofluidic fields^49^; however, all of the trapped cells are released at the same time. The characteristics of tunable noncontact trapping and the AST where only trapped cells were arranged one by one at each section of the tunnel make ‘quantum release’ of multiple trapped cells possible. First, we discussed the relationship between the number of trapped cells and the applied power and flow rate, as shown in Fig. 4d. The relevance of the capture capacity is similar to that of the capture efficiency, which increases as the power increases and decreases as the flow rate increases. The images in Fig. 4d show that as the power increases, SteAS dominated the movement of cells, and the trapping point expanded from a specific point to the entire AST, which is the direct reason for the increase in capture capacity. Moreover, the capture capacity and applied power at different flow rates show an excellent linear correlation. Based on this, the release of multiple cells was achieved digitally via tunable AST. When there are multiple trapped cells in the AST, the force analysis of cells at the trapping point is more complex than that of an individual trapped cell due to the interaction between cells. Under the action of lateral flow, the cells in the AST have a tendency to move toward the trapping point, as shown in Fig. 2f. Since the cells are arranged in the tunnel, the thrust on the cell at the trapping point will increase as the number of cells increases. When the thrust force is dominant, the cell at the trapping point will be pushed out of the tunnel and released downstream. The number of trapped cells is determined by the balance between the strength of the acoustic tunnel and the thrust effect. As shown in Fig. 4e and SI-Movie-9, different numbers of HeLa cells were trapped by SteAS at the beginning. Then, the cells were released one by one by gradually reducing the applied power. As a demonstration, we tried to controllably release two, three and four cells at a time. Ideally, by gradually reducing the power, the cell that enters the SteAST first would be released first at the fixed release point following a ‘first enter first release sequence’. However, in the actual experiment, due to the large initial power applied to prevent cell adhesion, the release sequence was not controlled enough. The strong streaming vortices caused the position exchange of cells during the trapping and release process. However, there is still an opportunity to control the sequence of release through optimization of power regulation, cell pretreatment and the shape of the device. Next, the combination of selective trapping and controllable release was also achieved as a proof of concept. With coordination of lateral flow, HeLa cells were extracted from diluted blood into a buffer and released through power regulation, as shown in Supplementary Fig. S13.

### Rare cell separation from undiluted blood by tuning the device angle

Thus far, we have systematically used the 1st and 2nd design principles to achieve size-based selective single-cell trapping, separation, and release. To improve the stability of separation in complex biological samples, we optimized the SteAST platform based on the 3rd design principle. Rare cells are low-abundance cells in a much larger population of background cells, such as CTCs, circulating fetal cells, and cells infected by a virus or parasite, which are highly important for various applications, such as liquid biopsy, prenatal diagnosis and identification of infection^73,74^. Various microfluidic strategies based on immune affinity^70^, microstructures^68^, hydrodynamics^71,75^, viscoelasticity and external fields^76^ have been attempted to achieve the separation of rare cells. In the SteAST system, the UHF BAW device is used to generate the AST, which selectively traps larger cells. Due to the excellent stability and biocompatibility for long-term trapping and the limited volume of the tunnel, it is actually suitable for rare cell separations. Here, we demonstrated rare cell separation directly from whole blood samples using SteAST. A major focus is on the effect of the relative tunnel position and angles to the microchannel.

Calcein-AM-stained HeLa cells were spiked into undiluted blood to mimic CTCs in the peripheral blood of cancer patients. CTCs are rare cells, with as few as one cell per 10^9^ blood cells in cancer patient blood, and have received tremendous research interest as emerging biomarkers for in vitro diagnosis^70^. Compared to diluted blood samples, undiluted whole blood is a more challenging sample. In view of the physical parameters, whole blood is more viscous and turbid. In addition, the high density of unwanted cells, especially red blood cells (10^9^/mL), may cause intense interactions among cells that change the trajectory of specimens and influence the stability of cell trapping. In the case of SteAS used in whole blood samples, the cells fall away from the vortices at the apex of the device, called the release points, as shown in Supplementary Fig. S14. The reason is that the continuity of the AST is interrupted at release points, which makes the cells more likely to separate. To decrease the negative effects induced by the cell–cell interactions, the relative angle between the UHF device and the microchannel was optimized to divide the trapping point and releasing points, as discussed in the 3^rd^ design principle in Fig. 2f. Using the previous structure, the release point was close to the trapping point, which facilitated the release of trapped cells, while the cell–cell interaction was violent. However, if we rotated the UHF device into symmetry along the lateral flow, then the trapping point was in the center of the edge near the downstream. In this case, the overlap of the trapping point and releasing point can be largely avoided. After this optimization, size-based separation of CTCs from whole blood was achieved, as shown in Fig. 4f and SI-Movie-10. The stacked images showed that CTCs were stably trapped in the trapping point, while the blood cells were released at the release points as designed.

### Cell manipulations by combining dynamic and quasi-static trapping

Based on the above discussion, the SteAST platform has two different working modes: quasi-static and dynamic. In quasi-static mode, the particles are trapped, arranged and rotated in a virtual tunnel under the action of torque induced by hydrodynamic forces. In the dynamic mode, lateral flow brings a tunable shearing effect and material transport effect. Combined with the unique handling characteristics, size-based selective cell trapping and controlled release of individual cells were successfully achieved in different samples. Next, we combined the above two operation modes and further demonstrated the online cell pretreatment, reaction and analysis.

**Fig. 5:**
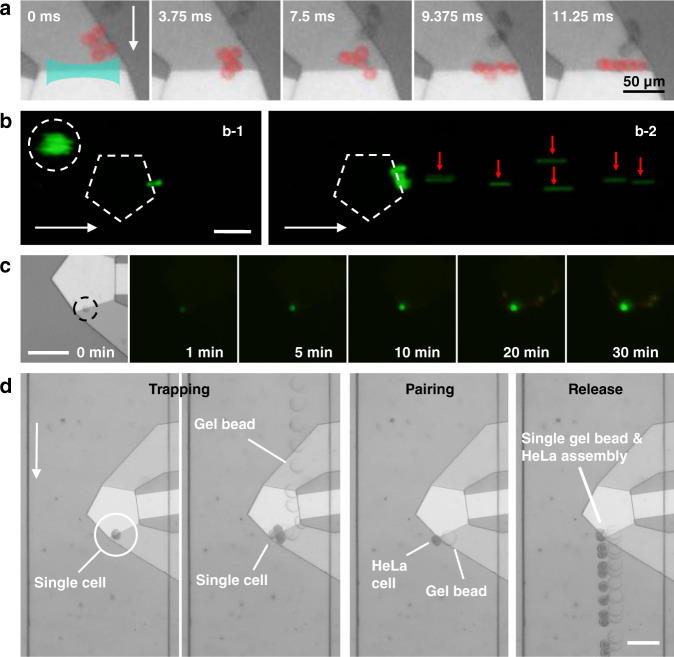
Dissociation and analysis of single cells. **a** Dissociation and arrangement of a cluster based on AST (blue zone). The cluster is dissociated into a single cell and aligned under the action of SteAS. The cells are highlighted by pseudocolor (red). The applied power is 66 mW, and the scale bar is 50 μm. **b** Dissociation of the cluster in continuous flow. (b-1) The HeLa cell cluster (dotted cycle) is attracted to the AST. (b-2) The trapped cluster is dissociated into individual cells (indicated by red arrows). The flow rate was 2 μL/min, and the applied power was approximately 15 mW. **c** In situ analysis of a single cell via SteAS. By switching between different lateral flow materials (HeLa cells and reagents), fluorescence-based analysis of trapped single cells is realized. **d** Pairing of a single cell and a barcode gel bead via AST. By switching between different lateral flow specimens (HeLa cells and gel beads), a single cell and a barcode gel bead are trapped in AST and contact each other at the trapping point. When the device is turned off, the assembled cell and bead are released together to move downstream. The white arrow points in the direction of lateral flow. The dotted pentagon represents the position of the UHF device. The scale bar is 100 μm.

Clusters are ubiquitous at the pretreatment step of various biosamples, including cultured cells and clinical samples, which often have a negative impact on manipulation or diagnosis. In single-cell manipulation and analysis platforms based on microfluidics, clusters are often the cause of microchannel blockage and a high multiplet rate. By the quasi-static mode of the single-cell-sized tunnel, the trapped clusters can be squeezed and dissociated in the tunnel, which provides a new strategy for preparing high-quality single-cell samples. When clusters entered the SteAST, because the cluster size was larger than the tunnel, the part outside the tunnel was sheared by acoustic streaming and squeezed by UHF BAWs. Finally, under the combined action of the two effects, the cell cluster was dissociated into individuals and arranged in the tunnel. This process was recorded by a high-speed camera and is shown in Fig. 5a. Compared to dissociation under quasi-static conditions, dynamic dissociation due to the expansion of the trapping point under the action of lateral flow makes the process slightly different. Under the action of lateral flow, the acoustic streaming vortices are compressed, while the acoustic wave area remains unchanged, which makes the equilibrium position of the particles change from a fixed point to a ring. This is the reason why the capture state of the particles changes from rotation to revolution. Even so, dynamic dissociation at the single-cell level is still achieved. Clusters of HeLa cells stained with calcein-AM were used to demonstrate this process. When a cluster was trapped in the SteAST, it was continuously dissociated into individual cells, as shown in Fig. 5b and SI-Movie-11. Compared with enzyme-based dissociation, the mechanical force-based method is more convenient and reliable without the need for centrifuge-based extraction and strict control of processing time. Compared with the filter-based pretreatment solution, SteAST provides an automatable technology that is suitable for processing samples in a small volume and integration with commercial analysis platforms, such as flow cytometry and high-throughput single-cell sequencing platforms.

Compared with cell trapping, online analysis of trapped cells is rather important since it can provide rich cellular information. The combination of precise control of the delivered materials in the dimension of time and space and contactless, label-free dynamic trapping brought by microfluidics and SteAST enables efficient online cell analysis. Herein, we introduce two different single-cell analysis modes: dye-based in situ single-cell analysis and sample preparation compatible with downstream commercial single-cell sequencing technologies.

Multiphase flow driven by syringe pumps was introduced into the system to achieve staining-based analysis of trapped cells. Herein, trypan blue (a vital stain used to selectively tag dead tissues or cells) was used as a probe to evaluate the viability of trapped cells, as shown in Supplementary Fig. S15. HeLa cells were trapped stably, and the cell membrane still had permanent selectivity after 560 s. The extraction processes were repeated three times to demonstrate the multistep analysis: Trypan blue buffer at t = 30 s, t = 313 s and t = 560 s and DMEM/PBS buffer at other times. Furthermore, with a fluorescence microscope and double stain kit (calcein-AM/propidium iodide (PI)), in situ analysis of single cells was achieved, as shown in Fig. 5c. By controlling the applied power precisely, a single HeLa cell was trapped. Then, calcein-AM and PI were injected into the microchannel. The trapped cells showed good viability after 30 min of trapping, which also illustrated the stability and biocompatibility of the SteAST system. This in situ analysis technology has good compatibility with different types of staining kits, which gives this platform the potential to be applied in the fields of cell subpopulation profiling, immunoassays and rapid detection of pathogens.

Among all single-cell analysis techniques, single-cell RNA sequencing (scRNA-seq) has become one of the most powerful approaches that enables unprecedented temporal and spatial resolution^77^. In recent years, high-throughput scRNA-seq has been established and commercialized by integrating droplet microfluidics technology and barcoded primer beads. At present, all three methods (inDrop, Drop-seq, 10X Genomics Chromium (10X)) use similar designs to generate droplets, on-bead primers with barcodes to distinguish individual cells, and unique molecular identifiers (UMIs) for bias correction. Although a process for encoding tens of thousands of cells has been realized, the process of pairing cells and barcode gel beads is random. For example, in Drop-seq, a probabilistic model of a barcode bead or a single cell packaged in a droplet conforms to Poisson distribution models, which allows only limited pairing efficiency^78^. Even the best-performing technology (10X) requires millions of barcode gel beads to complete the encoding of tens of thousands of cells. Moreover, in some cases, the number of cells is limited, and a loss of even one cell may affect the final result. This unobservable assembly method cannot guarantee that the target cells are successfully paired with gel beads. Based on this, we believe that the introduction of the SteAST platform to realize the observable and controllable pairing of barcode beads and cells can overcome the bottleneck of a low pairing rate and sample compatibility. Herein, the assembly of a single cell and a barcode bead (10X) was demonstrated as a proof of concept. Samples of HeLa cells and gel beads were diluted to ensure that only a single cell or gel bead passed through the main channel at a time. First, a single cell entered the main channel and was trapped by SteAS, as shown in Fig. 5d. Then, the pump for driving cells was turned off, while that for gel beads was turned on. A single barcode was injected into the main channel and trapped in the trapping point. The single HeLa cell and gel bead contacted each other at the trapping point to complete the pairing process. Under the action of the SteAST and lateral flow, an interaction between the single cell and the gel bead occurred, and then they were assembled together. When the assembly was achieved, the applied power was turned off, and the assembled cell and gel bead could be released for downstream analyses (to continue the standard protocol of 10X Genomics). The entire process is shown in SI-Movie-12.

### In situ analysis of CTCs from patient samples

**Fig. 6:**
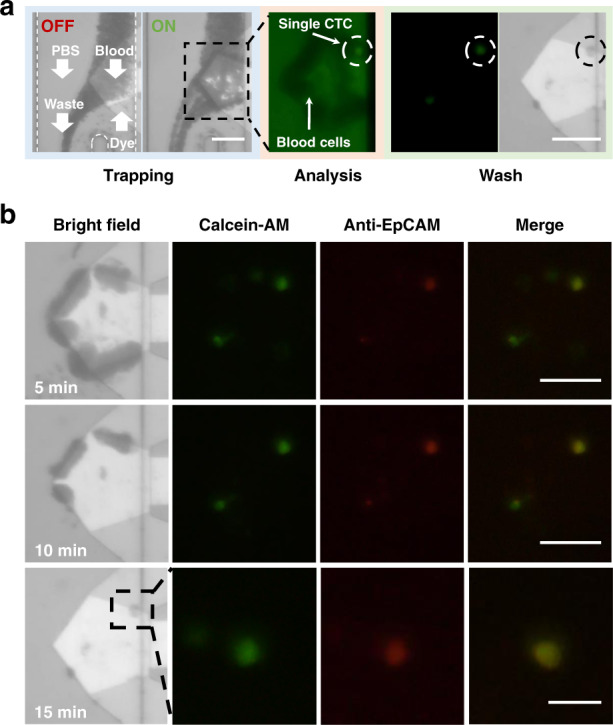
Separation and identification of CTCs from patient blood. **a** Process of single CTC separation from patient blood. The microchannel consists of three inlets and one outlet. The direction of flow is shown by white arrows. After CTCs are trapped in SteAS, dye is injected to achieve immune-based identification. The identified cells were washed to improve purity. **b** Process of staining and washing step. The trapped cells were stained green and red, which means that the cells demonstrated good viability and were CTCs from the perspective of immunology. The scale bars in the images and detailed images are 100 μm and 50 µm, respectively.

To further prove the potential of multimode manipulation based on SteAST in practical applications, we tried to separate and analyze CTCs in the undiluted blood of patients at the single-cell level. The heterogeneity and kinds of CTCs in patient blood are more complex and abundant than those in cultured cells, which makes CTC separation from patient blood more difficult than blood samples spiked with cultured cancer cells. This makes it difficult to obtain high-purity CTCs from the patient’s blood by separation methods based solely on size or immunity. Benefitting from the multimode handling mentioned above, we proposed a strategy for on-chip CTC separation from undiluted patient blood based on principles of both physics and immunity. The complete process is shown in Fig. 6a. First, the SteAST platform was used to selectively trap CTCs from the patient’s whole blood. Then, a fluorescently coupled antibody was injected into the microchannel to realize in situ immune-based analysis of the trapped cells. Finally, when the selectively trapped cells passed both physical and immunological identification, they were washed in situ to obtain a high purity and optionally released for subsequent analysis. Notably, due to the heterogeneity of CTCs in the actual patient, a higher strength of SteAST was used here to ensure the capture efficiency, and the final purity of CTCs was ensured at the washing step.

Stage IV lung cancer patient blood (*n* = 3) was chosen for these experiments. The dye mixed with calcein-AM and anti-epithelial cell adhesion molecule (anti-EpCAM)-labeled red fluorescence (Biolegend, USA) was injected into the channel to identify the type of trapped cell, as shown in Fig. 6a and SI-movie-13. EpCAM plays a role in the tumorigenesis and metastasis of carcinomas, so it can act as a diagnostic marker for various cancers and as a potential target for immunotherapeutic strategies^79^. The three-inlet (PBS buffer, blood sample and dye) and single-outlet microchannel was used here. When the power was applied, the blood cells were divided into two fluids by SteAST. After capturing the target cells, the inlets of blood and PBS buffer were closed, and dye was injected to identify the trapped cells. After staining, the dye inlet was closed, and PBS buffer was used to wash the remaining blood cells and dye. Calcein-AM (green) represents the viability and position of trapped cells, while anti-EpCAM-labeled red fluorescence represents EpCAM in the membrane of trapped cells. The results in Fig. 6b and Supplementary Fig. S16 show a trapped single CTC cluster from patient No. 1’s blood and 3T3 cell lines, which is a low-expression EpCAM cell line and was chosen as a target of the negative control group^80^. The results demonstrated that both CTCs and 3T3 cells were stained by calcein-AM and that CTCs were stained by anti-EpCAM, while 3T3 cells did not dye red due to the low expression of EpCAM, which indicates that the identification method is reliable. The procedures of in situ staining and washing are shown in Fig. 6b. After the single cell selectively trapped by SteAST was further identified as a CTC by immune recognition after 10 min, pure individual CTCs were obtained by power modulation and buffer washing. The results of selected trapped CTCs and clusters from patients No. 2 and No. 3 are shown in Supplementary Fig. S17.

## Discussion

In summary, starting from the basic theory, we discussed the relationship among acoustic streaming, acoustic fields and lateral flow through simulation to experiments to design a SteAST system for multimode manipulation at the single-cell level. Benefiting from the UHF BAWs in TE vibration mode and the small footprint of the device, highly confined SteAS is triggered by the attenuation of BAWs in liquid media. The scale of acoustic streaming vortices is tuned by microfluidics to adapt to the requirements for single-cell handling. Due to the UHF frequency, there is a short attenuation length (under 15 μm for 1.8 GHz), and thus the influence of standing acoustic waves can be ignored. Under the combined action of the acoustic radiation force and the drag force induced by vortices and lateral flow, a virtual tunnel is generated along the boundary of the UHF device, and quasi-static and dynamic modes are developed. Based on the two modes, basic cell manipulation is achieved, including selective trapping, rotation, dissociation, quantum release and pairing. SteAST is applicable over a range of lateral flow rates and can be rapidly tuned by applying power to adjust the strength of acoustic streaming. Notably, the migration and trapping processes in SteAST are repeatable without adhesion because the trapping point is suspended in the microchannel, which keeps trapped cells away from the device. To verify the possibility of multiple pretreatments and analyses in this platform, 3D reconstruction, separation, fluorescence-based in situ analysis and pairing with barcode gel beads for downstream analysis are demonstrated. Moreover, the selective trapping of CTCs in the undiluted patient’s blood based on both physical and immune detection is also achieved. Compared with other CTC separation technologies, SteAST can obtain extremely high purity (~100%) with high separation efficiency (Supplementary Table S1), and this two-dimensional identification based on physical and immunofluorescence avoids the bias of purely immune-based separation and the poor specificity in purely physical-based separation.

Although we have completed the preliminary verification of multimode handling, there are still many areas that need to be improved and problems that need to be resolved in actual applications. For example, in rotation-based reconstruction of a cluster, the image quality is poor, and the algorithm is rudimentary. In CTC trapping from whole blood, the fibers and detritus in the blood are entangled with the trapped cells under long-term processing, which affects the observation and subsequent quantum release process. Also, the inefficient and time-consuming pairing process relies on manual control. These problems can be optimized by system updates of both software and hardware in future work, including optimization of device shape, geometry of microfluidics and introduction of automatic fluid switching modules. At the same time, the platform has the potential to be developed. From the perspective of the platform itself, with a small footprint based on an IC-compatible process, the multi-UHF device integrated chip has the potential to be utilized in a multistep handling system and allows for easy integration with on-chip sensors for further characterization. Another advantage of the small footprint is that a large number of UHF devices can be integrated into one chip to improve the throughput and reduce costs. Limited by the performance-first design principle, the current shape of the UHF device is pentagonal, which makes the interaction between the tunnel and the lateral flow relatively fixed. From the perspective of the application, in conjunction with fluorescent image-based reconstruction, cells stained by kits can be subtyped and analyzed on a subcellular scale from both 3D bright fields and fluorescent fields. In addition, the existing dissociation procedure causes the loss of spatial information that is necessary for reconstructing the spatial organization of single-cell genomic and transcriptomic landscapes in a cluster/tissue. Based on a single-cell-scale tunnel, if the manipulation of 3D reconstruction, visualized dissociation and controllable release are integrated, then there is an opportunity to convert the spatial position information of each cell into time sequence information to achieve the dissociation of clusters without losing spatial information.

Previous studies based on UHF BAWs in microfluidics chips have mainly focused on the enrichment effect of particles. When SteAST interacts with biological particles, due to the heterogeneity of sizes and morphology, the characteristics are highlighted, including the effects of rotation, 3D focusing, and dissociation, which make multimode manipulation for complex applications in biological research possible. Altogether, we believe this platform, as a versatile tool, shows great potential as a μ-TAS or pretreatment module to be integrated into existing single-cell analysis processes for applications in liquid biopsy, personalized cancer therapy, drug discovery and high-throughput scRNA-seq.

## Materials and methods

### Design and fabrication of the device

The UHF BAW resonator is designed and fabricated by simulation software and IC-process. More details are provided in the SI.

### System setup

The acoustic resonator was controlled by a sinusoidal signal (1.8 GHz), which was generated by a signal generator (Agilent, N5171B) and amplified by a power amplifier (Mini-Circuits, ZHL-5 W-422+). The resonator was wire-bonded to evaluation boards for signal transmission. The performance of the UHF device was tested by a vector network analyzer (Agilent, N50171C). The PDMS channel was fabricated by a standard soft lithography process and assembled with the silicon substrate by pressure. The inlet and outlet holes in the PDMS channel were created by a syringe needle. The sample was driven by a syringe pump (Harvard, 70-4504). The syringe was connected to a microchannel by a Teflon tube (the inner diameter was 0.3 mm). The signal generator and syringe pump were controlled by LabVIEW software. The experiments proceeded on the stage of a fluorescence microscope (Olympus, BX53) with a CCD camera (Olympus, DP73) or a high-speed camera (Photron, UX50) and confocal microscope (Leica, SP8).

### Finite element simulation

A 3D model of the UHF device was built in COMSOL Multiphysics 5.5. (COMSOL Inc., USA)^81^. More details are provided in the SI.

### Sample preparation

PS particles (Macklin) were diluted with DI water and sonicated for 5 min to improve the monodispersity. HeLa cells and 3T3 cells were grown in Dulbecco’s modified Eagle medium (DMEM) supplemented with 10% fetal bovine serum and 1% penicillin–streptomycin in an incubator at 37 °C and 5% CO_2_, followed by dissociation with trypsin. Then, suspended cells were extracted in isotonic phosphate-buffered saline (PBS) solution by centrifugation (500 × *g*, 6 min for HeLa and 800 × *g*, 4 min for 3T3). Ethics approval for sample collection was approved by Tianjin Medical University Cancer Institute & Hospital Ethics Committee (E2016055). Blood from healthy donors and patients was acquired from Tianjin Medical University Cancer Institute & Hospital. Blood samples were collected in vacutainer tubes containing anticoagulant ethylenediaminetetraacetic acid (EDTA) and processed within 2 days. For the experiment of CTC separation from diluted blood, HeLa cells were trypsinized and resuspended at the desired concentration (1 × 10^5^cellsper mL) in PBS buffer, added to human blood samples, diluted 100-fold with PBS buffer (10~100 HeLa cells/10^4^ red blood cells) and mixed for 10–15 min at room temperature. For CTC separation from the patient’s blood, undiluted whole blood was injected into the microchannel.

### 3D reconstruction

The 3D reconstruction procedure can be divided into image processing and reconstruction. More details are provided in the SI.

## Supplementary information

Supplemental Material

SI-Movie-1_Simulation results of focusing and trapping via SteAST

SI-Movie-2_Simulation results of focusing and trapping via rotated SteAST

SI-Movie-3_Quasi-static trapping observed by confocal microscopy (xzyt mode)

SI-Movie-4_Quasi-static trapping observed by confocal microscopy (xzt mode)

SI-Movie-5_Trapping and arrangement of individual cells via SteAST

SI-Movie-6_Rotational manipulation of clusters recorded by a high speed camera

SI-Movie-7_Focusing and trapping of single cells

SI-Movie-8_Simulation results of particle separation

SI-Movie-9_Quantum release

SI-Movie-10_Separation and extraction of CTCs from whole blood

SI-Movie-11_Continuous dissociation of clusters

SI-Movie-12_Assembly of a single cell and a barcode gel bead

SI-Movie-13_Separation and immune identification of CTCs from patient blood

## Data Availability

All data are available in the main text or the supplementary materials. Further information is available from the corresponding author upon reasonable request.
