# Supplementary material for: Manipulation of single cells via a Stereo Acoustic Streaming Tunnel (SteAST)
Source: Microsyst Nanoeng. 2022 Aug 4;8:88. doi: 10.1038/s41378-022-00424-9 (PMC9352906; doi:10.1038/s41378-022-00424-9)
Supplement: Supplementary file 1 — Supplemental Material [file 41378_2022_424_MOESM1_ESM.docx]

Supplementary Materials for

**Manipulation of Single Cells via a Stereo Acoustic Streaming Tunnel (SteAST)**

Yang Yang, Wei Pang, Hongxiang Zhang, Weiwei Cui, Ke Jin, Chongling Sun, Yanyan Wang, Lin Zhang, Xiubao Ren, Xuexin Duan*

*Corresponding author. Email: xduan@tju.edu.cn

**This PDF file includes:**

Figs. S1 to S18

Movies S1 to S13

Table. S1

**Other Supplementary Materials for this manuscript include the following:**

Movies S1 to S13

**Design and fabrication of the device.** UHF BAWs were generated by applying an alternating current (AC) signal across the piezoelectric layer that was grown by aluminum nitride (AlN) (65). Due to the inverse piezoelectric effect, a spatial gradient in electrical potential results in mechanical displacement in the case of a piezoelectric material. The parallel resonant frequency of the UHF BAW device can be approximated by:

$$f_{a,n}=\left( 2n+1 \right)\cdot\frac{v^{D}}{4d},n=0,1,2,\ldots$$

where f_a,n_ is the n-th order parallel resonant frequency of the UHF device, n is the number of orders, v^^D^ is the acoustic velocity within the piezoelectric layer and d is the half thickness of the piezoelectric film. The applied frequency of the UHF device was the parallel resonant frequency in the 0th order. The thickness of the piezoelectric layer, which determines the resonant frequency of the BAW device, was controlled by the process of film deposition. The deposition thickness could be tuned from several nanometers to micrometers, so the resonant frequency of UHF BAW covers hundreds of megahertz to tens of gigahertz (60).

For the Bragg reflector, AlN and silicon dioxide (SiO_2_) were chosen. For the BE layer, compared with gold (Au), molybdenum (Mo) has better adhesion, which is important for the subsequent growth of the piezoelectric layer. For the piezoelectric layer, AlN has the largest wave velocity among mainstream piezoelectric materials and is compatible with the CMOS process, which makes it very suitable as a piezoelectric material for GHz acoustic devices (66). For the TE layer, Au is an ideal electrode material and widely used in the field of integrated circuits. Au electrode can be compatible with CMOS packaging technology (wire bonding), which is the way that our device connects with peripheral circuits.

The actual image, SEM image and structure of the GHz BAW device is shown in Supplementary Fig. S1a, b and c. The differently colored regions in the Fig. S1b show the different patterned electrodes, and the outlines of the bottom electrode (BE) and top electrode (TE) are depicted with green and yellow dashed lines, respectively. The Bragg reflector layer and the piezoelectric layer cover the entire silicon substrate. The area where the top and bottom electrodes overlap is the actual vibration area of the UHF BAW device. Briefly, from bottom to up, the Bragg reflector consisting of three alternating layers of silicon dioxide (SiO2) and aluminum nitride (AlN) was deposited on the silicon substrate, then, BE (molybdenum (Mo), 600 nm), Piezoelectric layer (AlN, 1000nm) and TE (Gold (Au), 800nm) were deposited and patterned on the Bragg reflector to form a sandwich structure for acoustic vibration. The active area of the resonator is approximately 0.01 mm^2^ and patterned as a pentagon to achieve highly efficient energy transduction.

**Finite Element Simulation:** To evaluate the SteAST and trajectories of particles in acoustic vortices, a 3D model of the UHF device was built in COMSOL Multiphysics 5.5. (COMSOL Inc., USA). Gigahertz vibrations were triggered by the inverse piezoelectric effect. The stereo acoustic streaming induced by vibration was governed by a decaying body force generated from acoustic attenuation. The UHF device in the simulation had the same size and geometry as the experimental device. The liquid material was set as water, and the velocity field can be described by the incompressible Navier–Stokes equation. In the 3D simulation, radiation force was given in an area with a pentagon-shaped bottom (side length 120 µm) and 50 µm height. Particles flowing through the UHF device were governed by the drag force induced by SteAS and the lateral flow and radiation force triggered by attenuated acoustic pressure.

**3D Reconstruction.** The 3D reconstruction procedure can be divided into image processing and reconstruction. Briefly, (1) The image sequence with the boundary of the UHF device as the center was cropped into a suitable size. (2) The dark background was removed by judging the gray value. (3) Cells in the silicon area were identified by comparison with the gray value of the background, and their gray value was multiplied by the coefficient P (P=0.85, Supplementary Fig. S10) to enhance the contrast. In the 3D reconstruction, since the cell surface information is concealed in the dark background part, only the white background part was used for modeling. The reconstruction procedure is briefly summarized as follows: (1) The position of the rotation axis was obtained by overlapping two pictures with a phase difference of π, which coincides with the boundary of the UHF device. (2) The image was cropped with the rotation axis as the boundary, and the white background part was retained. (3) The relative angle of each image was calculated as follows:

$$\alpha_{n}=2\pi*\frac{n}{N}$$

where α_n_ is the relative angle of n-th images in the sequence. N and n are the number of images for one rotation cycle and the sequence number of the currently processed image, respectively. (4) The average gray value of the image was taken as a threshold to identify the background and cells and extract the information of cells by subtracting the background. (4) The following formulas were used to obtain the spatial position of the pixel in each picture through coordinate transformation:

$${x'}_{n}=x_{n}*\cos\alpha_{n}$$

$${y'}_{n}=y_{n}$$

$${z^{'}}_{n}=x_{n}*\sin\alpha_{n}$$

where (x_n_, y_n_, α_n_) and (x’_n_, y’_n_, z’_n_) are the spatial positions before and after coordinate transformation, respectively. The schematics of reconstruction are shown in Supplementary Fig. S18.


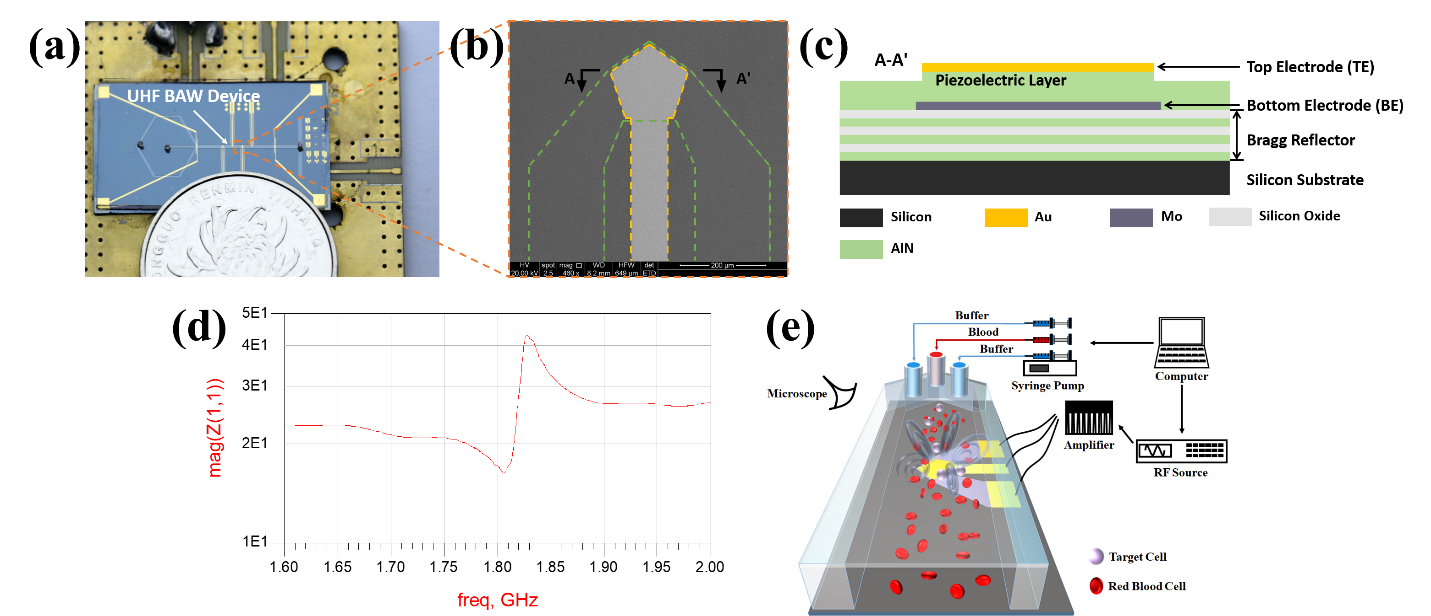
Fig. S1. The images and structures of GHz BAW device and schematic of stereo acoustic streaming tunnel (SteAST) system.

(a) An actual image of SteAST platform, which is consisted by ultrahigh frequency (UHF) bulk acoustic waves (BAW) device on silicon substrate and polydimethylsiloxane (PDMS)-based microchannel. There is a one-yuan coin for scale. (b) SEM image of GHz acoustic device. The BE and TE layer are highlighted by green and yellow dashed lines. (c) Structures of UHF BAW device in section view. The device is consisted by silicon substrate, Bragg reflector, BE, piezoelectric layer and TE from bottom to up. (d) Representation of resonant frequency of UHF device. The frequency spectrum of UHF device shows the resonant frequency of UHF device is about 1.8 GHz. (e) Schematic of SteAST system. The system is consisted by a PDMS microchannel, silicon substrate where an UHF-BAW device is integrated, syringe pumps for fluid driving, a RF source and an amplifier for the excitation of UHF BAWs and a computer for control.


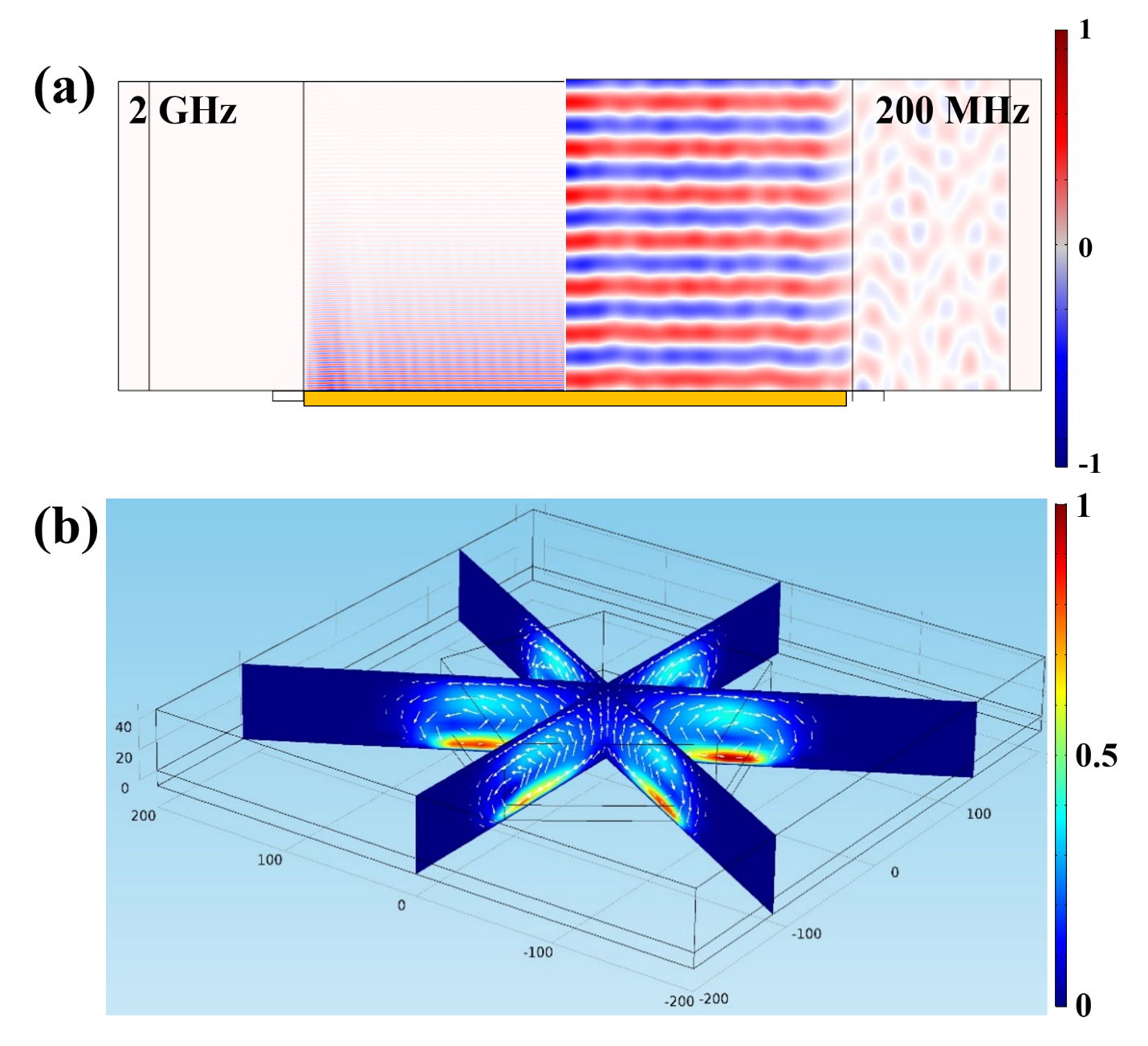
Fig. S2. Representation of ultrahigh-frequency (UHF) device and stereo acoustic streaming (SteAS).

(a) Finite element simulation results of attenuation of acoustic wave at high (2 GHz)/low (200 MHz) resonant frequency. The UHF device is represented by the yellow rectangle. (b) 3D finite element simulation results of SteAS without lateral flow. The white arrows point the direction of streaming. The color bar shows the normalized velocity of streaming in the microchannel (50 μm).


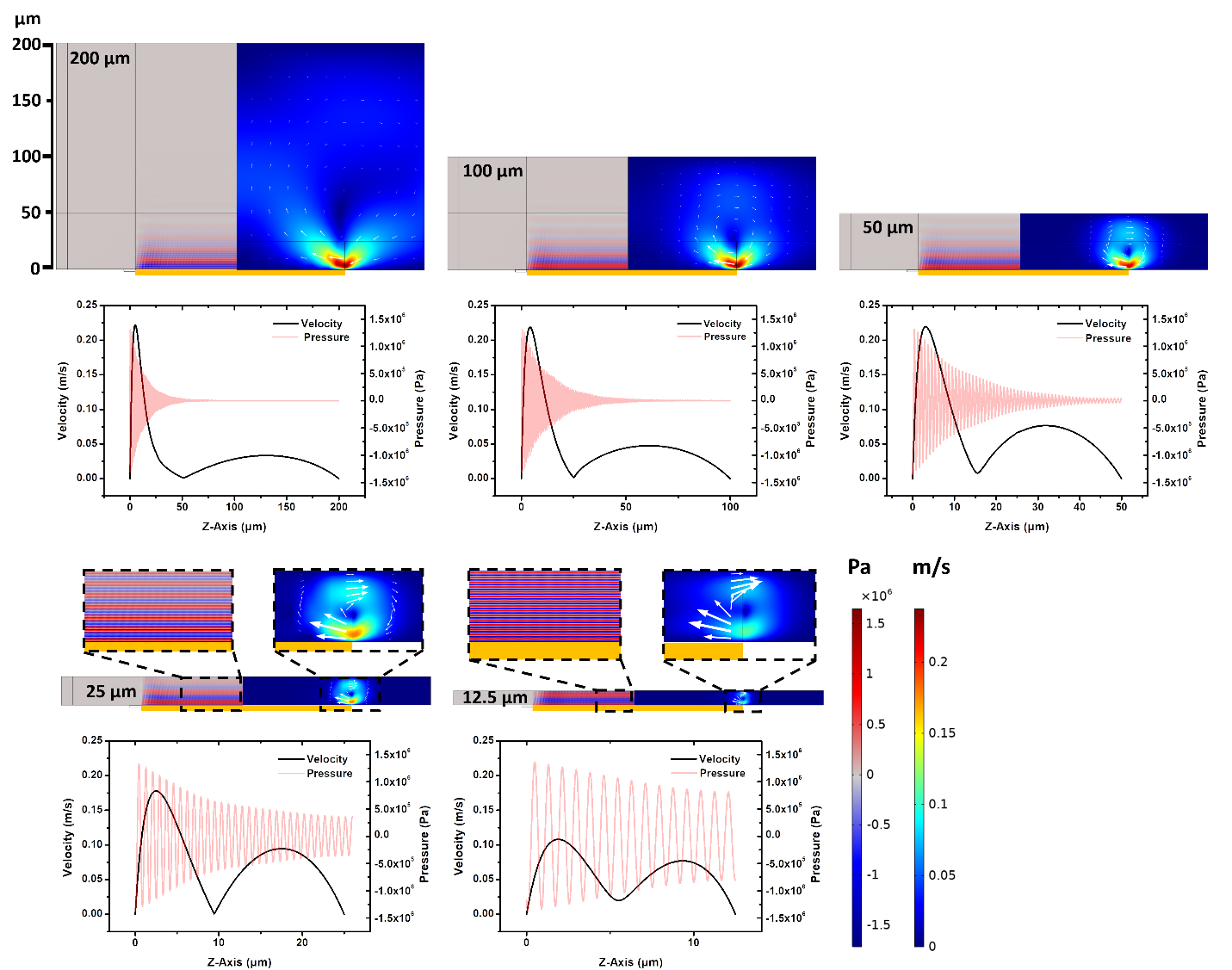
Fig. S3. Distribution of acoustic waves and acoustic streaming in different height microchannel.

The simulation results of distribution of acoustic waves and the acoustic vortex in microchannel with height of 200, 100, 50, 25, 12.5 μm. The graphics below the images shows the strength distribution of the two in z-direction. The pressure of acoustic waves and the velocity of the vortex are extracted from the center and boundary of device, respectively.


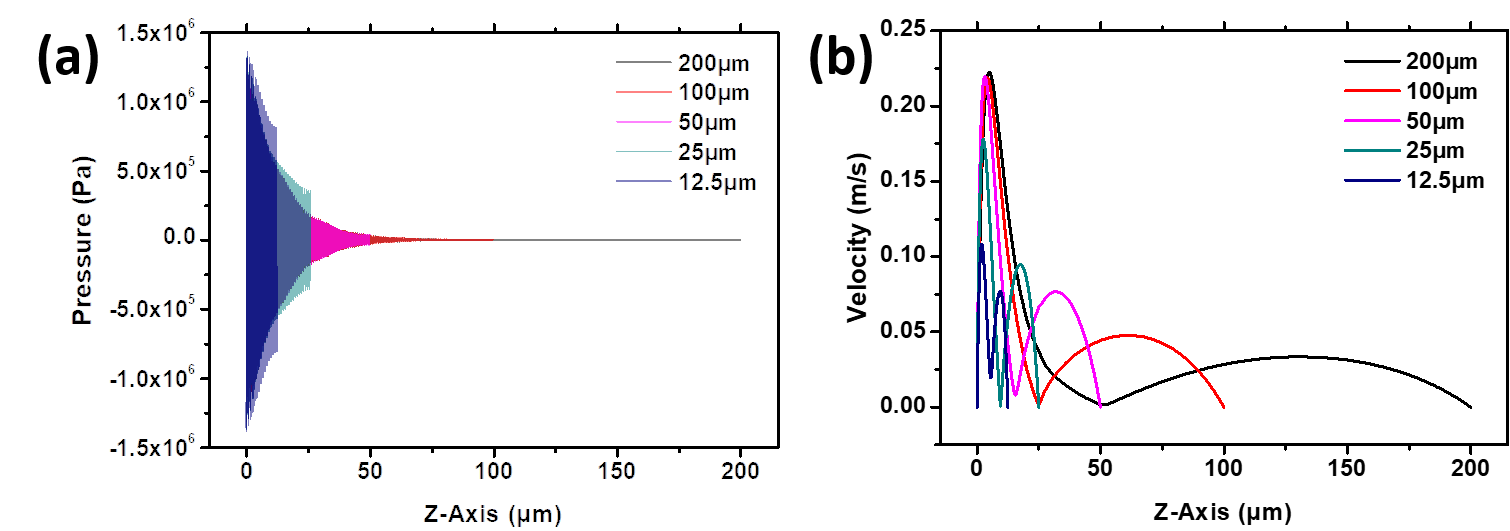


Fig. S4. Distribution of acoustic waves and acoustic streaming in height direction.

(a) The distribution of the acoustic field in the height direction under different heights of microchannel at the center of the device. (b) The distribution of the velocity of acoustic vortex in the height direction under different heights of microchannel at the boundary of the device.


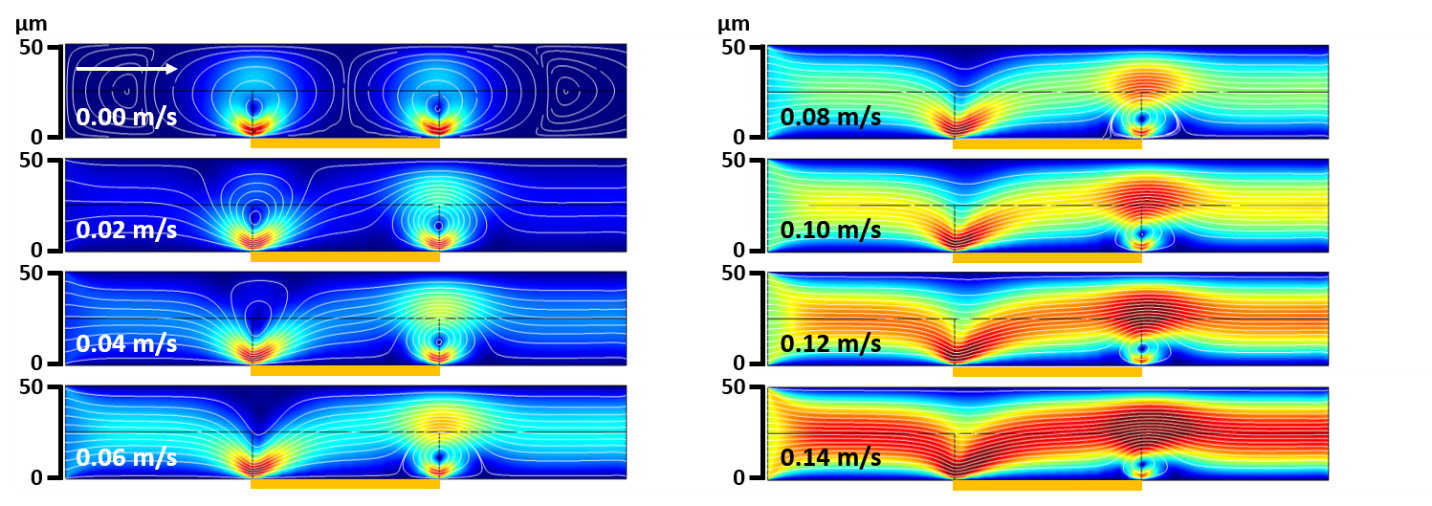


Fig. S5. Distribution of acoustic streaming in different flow rate of lateral flow.

The distribution of acoustic vortices changes with the increase of lateral flow velocity. The direction of lateral flow is pointed by the white arrow. The position of UHF device is highlighted by yellow rectangles.


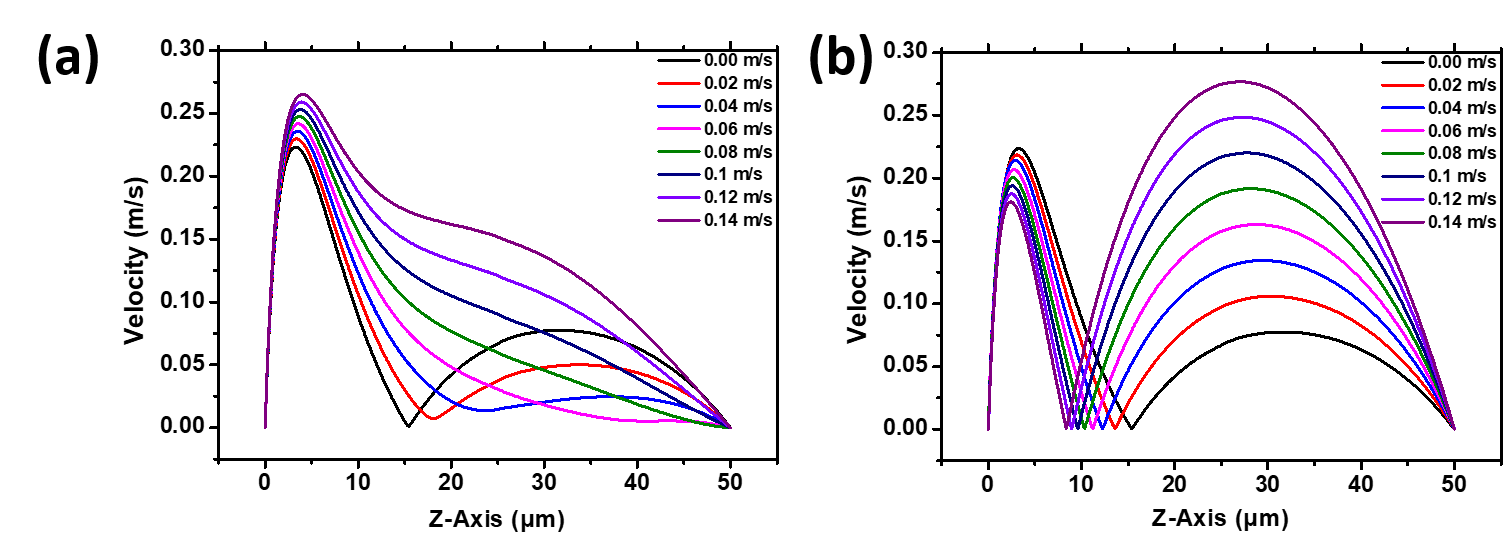


Fig. S6. Distribution of acoustic streaming in height direction.

(a) The distribution of flow velocity in z-axis at the upstream boundary of device with lateral flow in different velocity. (b) The distribution of flow velocity in z-axis at the downstream boundary of device with lateral flow in different velocity.


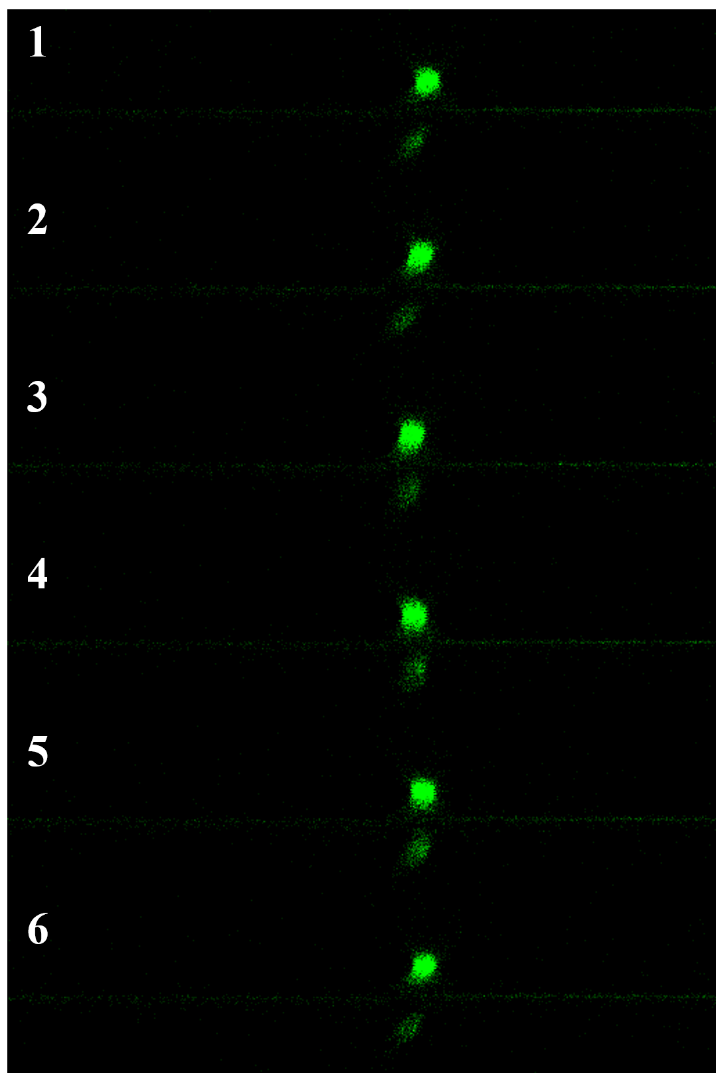


Fig. S7. Rotational manipulation of a single particle in acoustic vortices.

The position in x-z-plane is captured by confocal microscope via x-z-t mode. Continuous images in one rotation cycle is shown. The number represents the shooting order. The time interval between pictures is 27 ms.


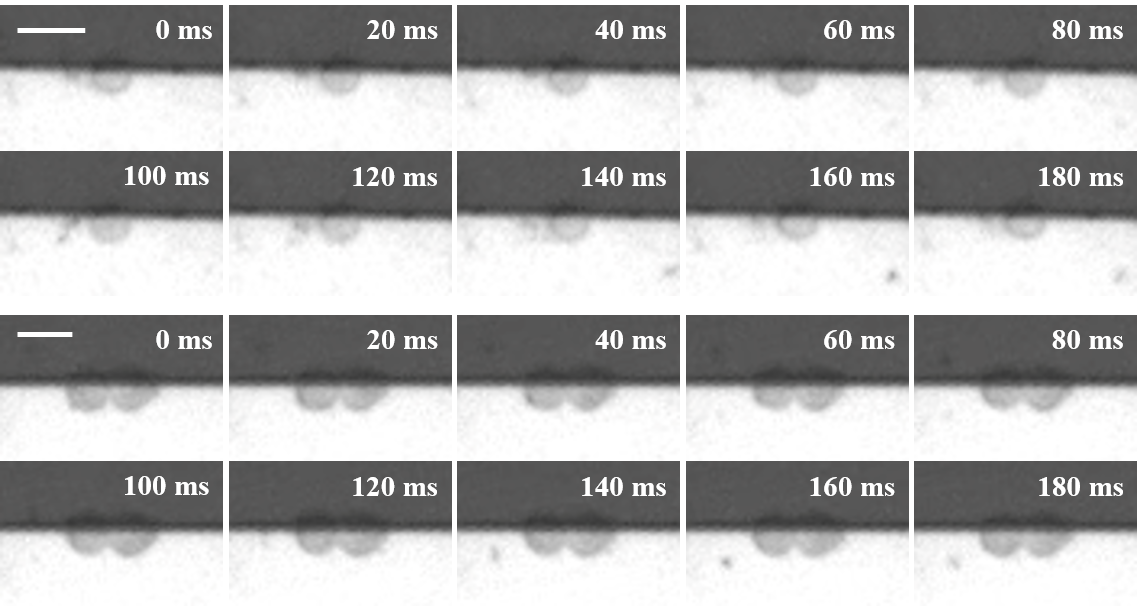


Fig. S8. Rotation of a single cell and dimer of cells.

The rotation process of a single HeLa cell and a dimer shot by the high speed camera in 180 ms. Scale bar is 25 μm.


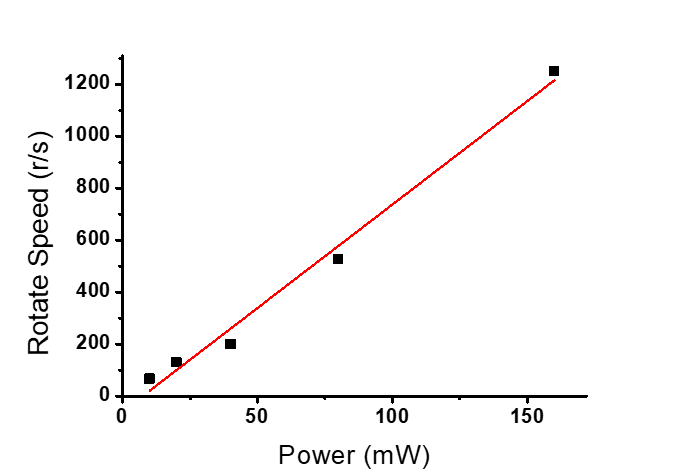


Fig. S9. Representation of rotation speed with the applied power.

The relationship between the applied power (mW) and rotation speed (rotation per second). And the red line is the results after the linear fit.


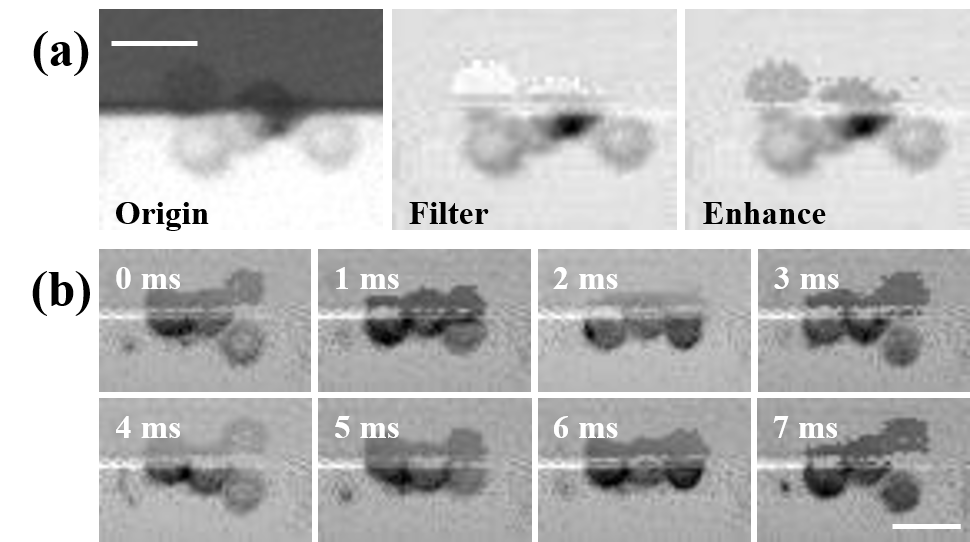


Fig. S10. Image processing.

(a) The background of original images is removed by software (MATLAB). (b) The gray value in zone of cells is enhanced. Scale bar is 25 μm.


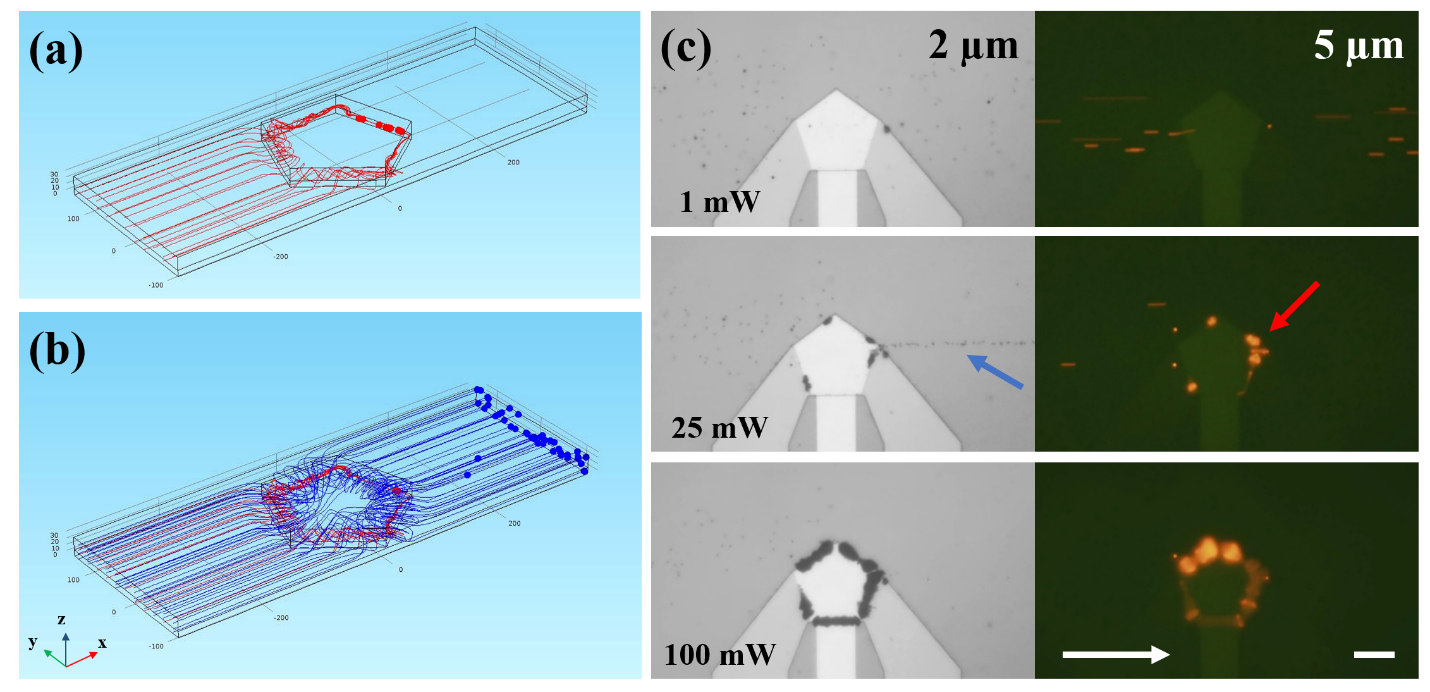


Fig. S11. Size-based selective trapping of particles.

The simulation results of particle (a) trapping and (b) separation. The trajectories of particles are shown by red and blue lines. The red particles (15 µm) are trapped in the edge of device while the blue particles (2 µm) escaped. The trajectories of red particles are obviously closer to the centers of vortices than trajectories of blue particles. (c) Power-tuned selective trapping of PS micro-particles. When the applied power is low (1 mW), both 5 μm particles and 2 μm particles can’t be trapped by UHF device. When the applied power is suitable, the size-based separation was achieved. The 5 μm particles were trapped while the 2 μm particles were go through the acoustic streaming tunnel. when the applied power is high (100 mW), both the 5 μm particles and the 2 μm particles were trapped in the tunnel.


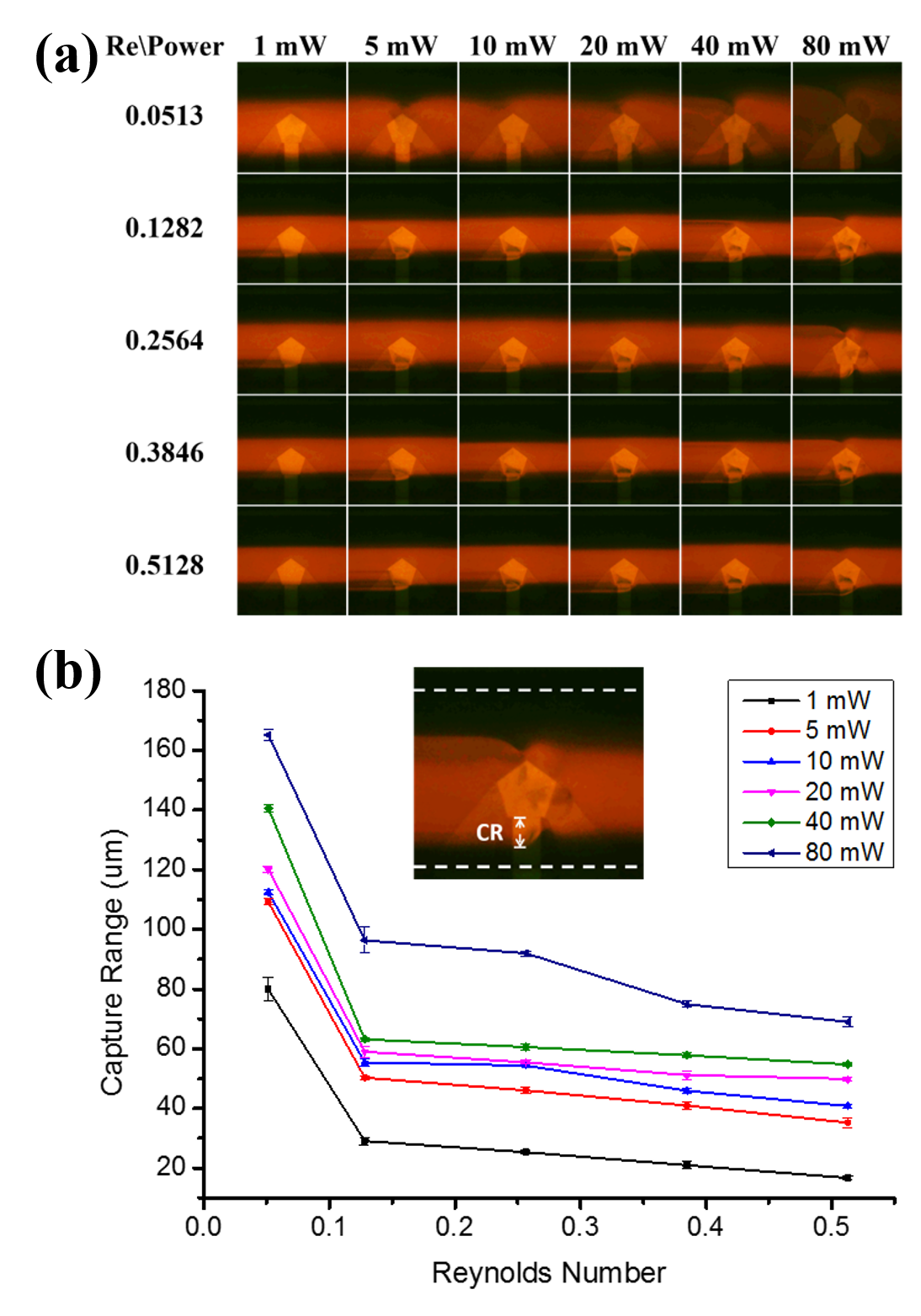


Fig. S12. Actuating range (capture range) of SteAS.

(a) The range of steaming were represented by the red fluorescent dye. (b) The range of streaming, defined as capture range (CR), were quantized. The relationship among CR, applied power and lateral flow rate (Reynolds number) were demonstrated.


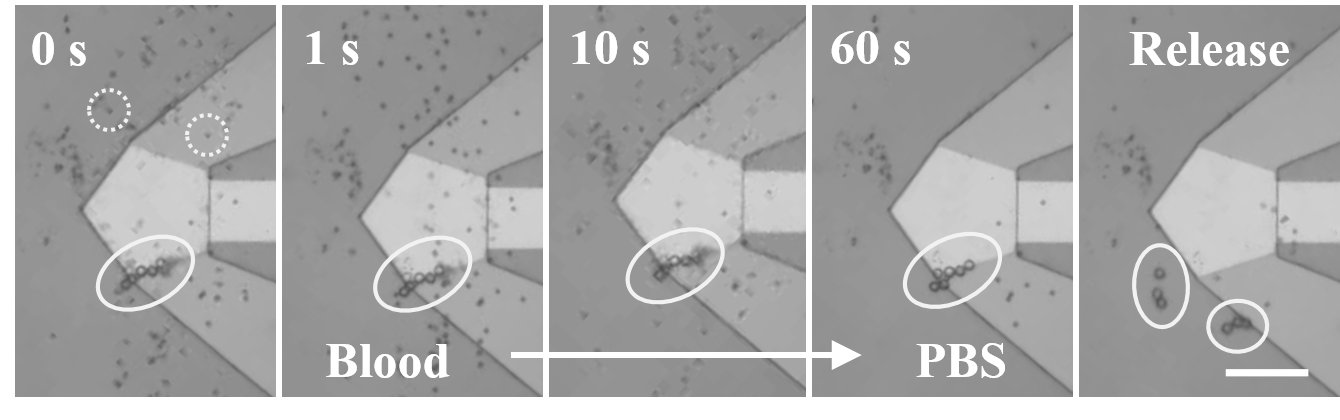


Fig. S13. Extraction and controllable release of trapped cells via the SteAST platform.

HeLa cells in diluted blood are extracted from blood samples to PBS buffer. After the extraction, the CTCs are released for downstream analysis in PBS buffer by turning off the device.


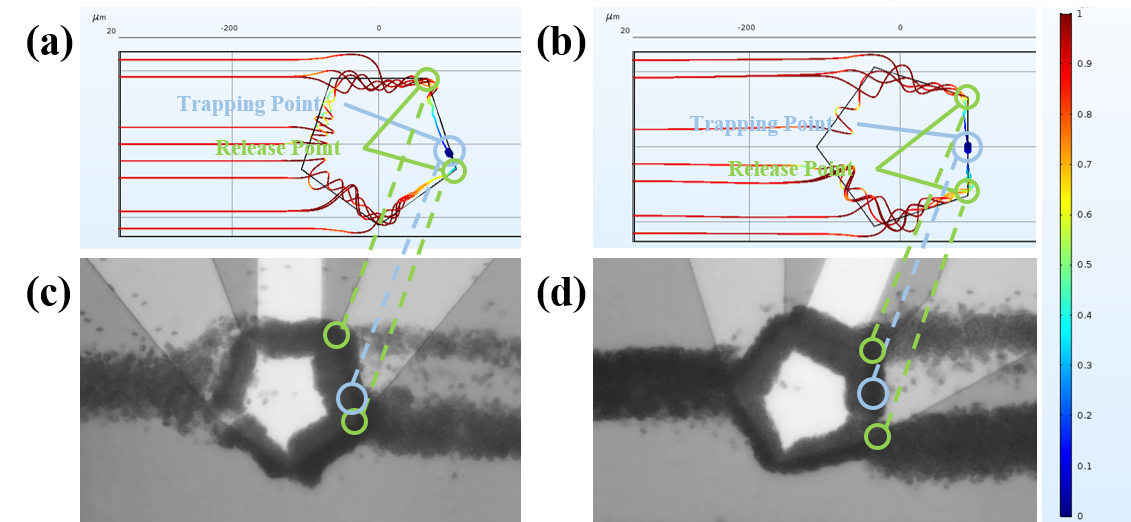


Fig. S14. Optimization of the angle between the UHF device and lateral flow.

By adjusting the relative angle between the device and the lateral flow, the distance between the release points and the trapping point is lengthened. Simulation (a)/(b) and experimental (c)/(d) results before/after optimization. The trapping point and release points are highlighted by blue and green cycles, respectively. The color bar shows the normalized speed of particles.


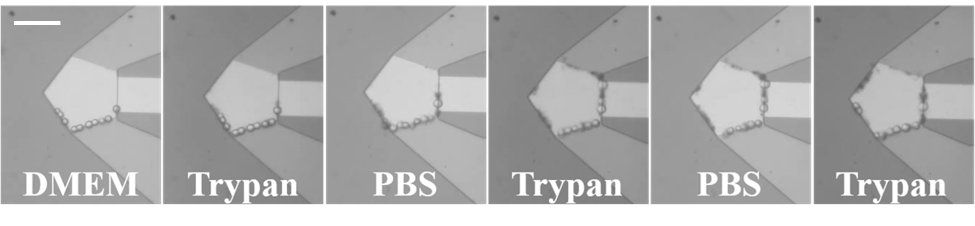


Fig. S15. Viability analysis of trapped cells via the SteAST platform.

Trypan blue was utilized to test the viability of trapped HeLa cells. Trapped cells were extracted from buffer (DMEM/PBS) with trypan blue for three cycles. The trapped cells were in good viability after being stimulated for 560 s. The scale bar is 100 μm.


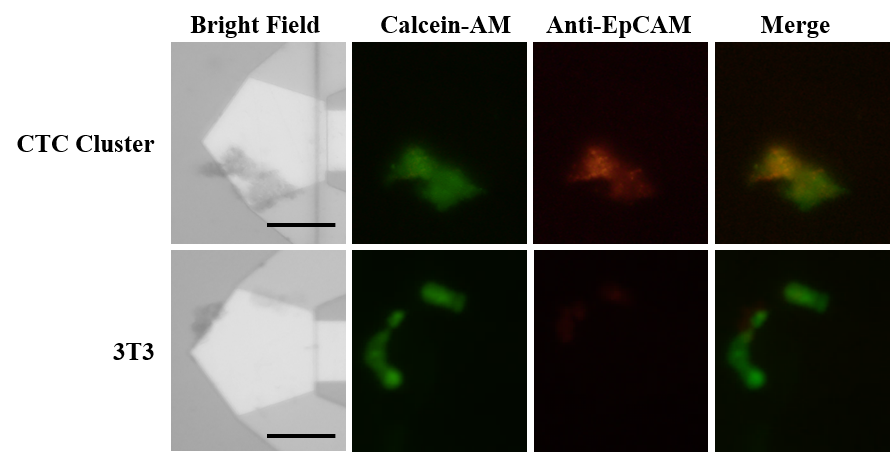


Fig. S16. In situ analysis of CTC clusters and cultured cells (3T3).

Images of a trapped cell cluster and 3T3 cells (low expression of EpCAM) stained with calcein-AM and antibodies against EpCAM on a chip. Scale bar is 100 μm.


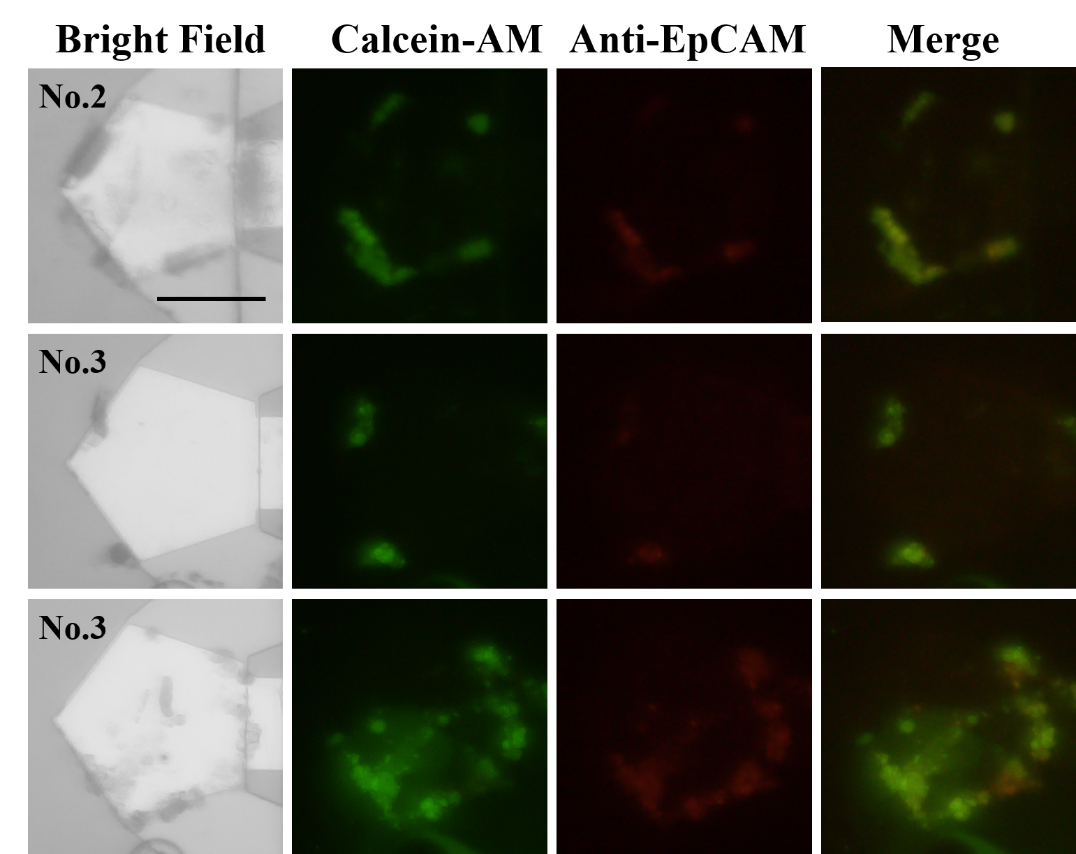


Fig. S17. On-situ analysis of CTCs from patients.

Images of trapped CTCs from patients (No. 2 and No. 3) stained with calcein-AM and antibodies against EpCAM. The scale bar is 100 μm.


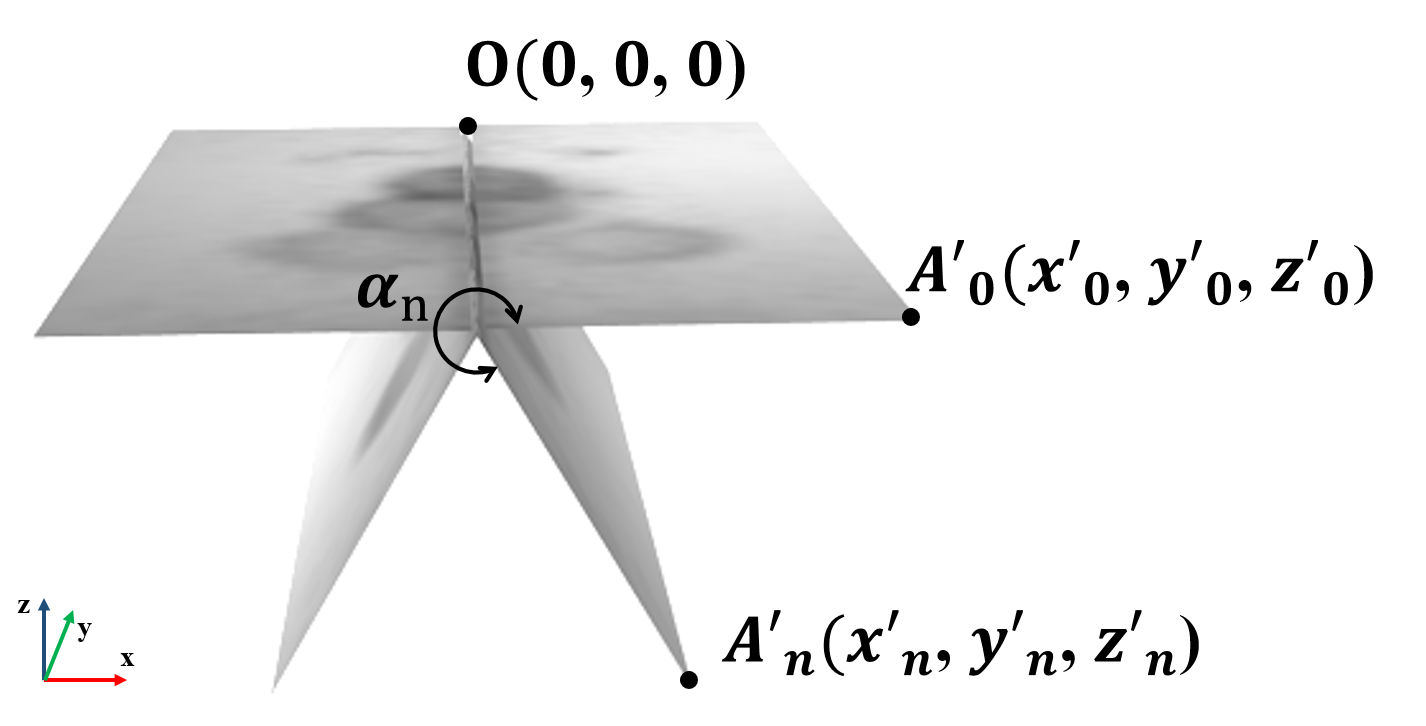


Fig. S18. 3D reconstruction of clusters.

Initialization of the 3D grid of voxels according to 2D image sequencing. The space coordinates are obtained by the space transformation from the polar coordinate system to the Cartesian coordinate system. The angle in the polar coordinate system is calculated by the image number in a cycle.

Table 1 Comparison of different CTC separation techniques


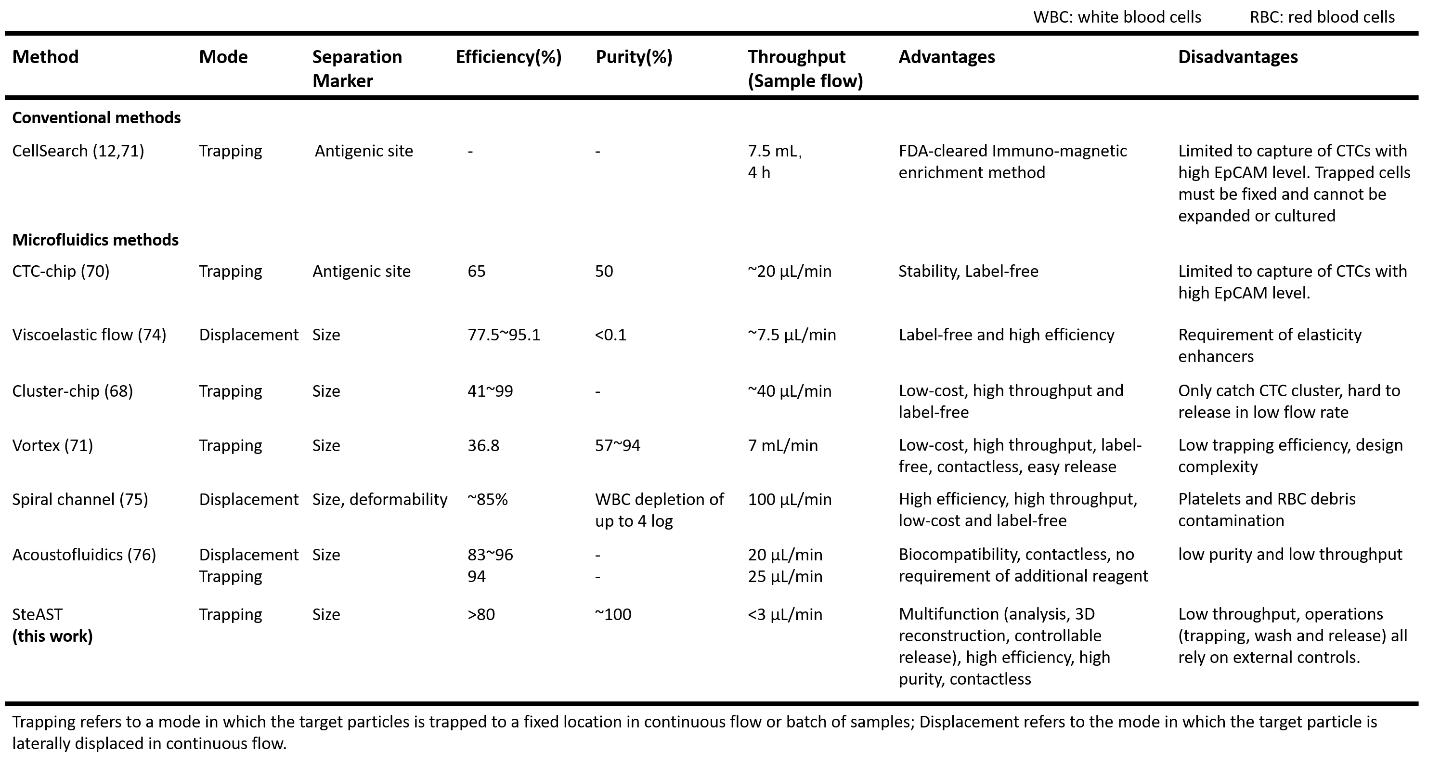


Movie S1.

Simulation results of focusing and trapping via SteAST.

Movie S2.

Simulation results of focusing and trapping via rotated SteAST.

Movie S3.

Quasi-static trapping observed by confocal microscopy (xzyt mode).

Movie S4.

Quasi-static trapping observed by confocal microscopy (xzt mode).

Movie S5.

Trapping and arrangement of individual cells via SteAST.

Movie S6.

Rotational manipulation of clusters recorded by a high speed camera.

Movie S7.

Rotational manipulation of clusters recorded by a high speed camera.

Movie S8.

Simulation results of particle separation.

Movie S9.

Quantum release of trapped cells by tuning the applied power.

Movie S10.

Separation and extraction of CTCs from whole blood.

Movie S11.

Continuous dissociation of clusters.

Movie S12.

Assembly of a single cell and a barcode gel bead.

Movie S13.

Separation and immune identification of CTCs from patient blood.
